# Transferrin receptor 1 binds human parvovirus B19 VP1u to facilitate entry

**DOI:** 10.1038/s41467-026-74283-7

**Published:** 2026-06-11

**Authors:** Hyunwook Lee, Jan Bieri, Nicolas Ammann, Corinne Suter, Daniela Hunziker, Ajit K. Singh, Carol M. Bator, Susan L. Hafenstein, Carlos Ros

**Affiliations:** 1 The Hormel Institute, University of Minnesota, Austin, MN USA; 2 Department of Chemistry, Biochemistry and Pharmaceutical Sciences, University of Bern, Bern, Switzerland; 3 Graduate School for Cellular and Biomedical Sciences, Bern, Switzerland; 4 Department of Biochemistry, Biophysics and Molecular Biology, University of Minnesota, Minneapolis, MN USA; 5 Department of Infectious Disease, Mayo Clinic, Rochester, MN USA

**Keywords:** Microbiology, Virus-host interactions, Structural biology

## Abstract

Human parvovirus B19 (B19V) displays a strict tropism for erythroid progenitor cells, which is governed by the VP1 unique domain (VP1u). This domain mediates cell-specific uptake through interaction with an unknown cellular receptor, termed VP1uR. Proximity labeling in permissive erythroid cells identifies transferrin receptor 1 (TfR1/CD71) as a predominant membrane protein associated with VP1u. VP1u constructs colocalize with TfR1 at the cell surface of erythroid cells. Incubation with anti-TfR1 antibody OKT9 abolishes binding and uptake of recombinant VP1u. While OKT9 efficiently inhibits B19V uptake and infection, it does not block virus binding to host cells. Direct binding assays confirm interaction of VP1u with human TfR1. Using cryo-EM we solved the 2.4 Å structure of the TfR1-VP1u complex, mapping the binding site. These findings establish TfR1 as the previously unknown receptor, VP1uR, required for B19V uptake.

## Abstract (web summary)

Researchers identify transferrin receptor 1 as the long-sought receptor, previously termed VP1uR, required for parvovirus B19 entry into erythroid cells, and determine the cryo-EM structure of the receptor-virus interaction.

## Introduction

Human parvovirus B19 (B19V), the prototype member of the genus *Erythroparvovirus*, is a common human pathogen associated with a range of clinical manifestations, including erythema infectiosum, transient aplastic crisis, chronic anemia in immunocompromised individuals, and severe fetal disease following intrauterine infection^1^. A defining feature of B19V biology is its exceptionally narrow cellular tropism: productive infection is restricted exclusively to erythroid progenitor cells (EPCs) in bone marrow and fetal liver^2,3^. This stringent restriction is a central determinant of viral pathogenesis.

Early studies identified the glycosphingolipid globoside as the cellular receptor for B19V^4^. However, globoside expression is not restricted to erythroid cells, and subsequent work demonstrated that globoside alone cannot account for the selective uptake and tropism of the virus^5^. Genetic and functional analyses showed that globoside is dispensable for viral uptake but essential at a post-entry step required for productive infection^6^. Recent work established globoside as a conditional receptor whose function is controlled by the local pH conditions^7^. At the mildly acidic airway mucosa, B19V binds globoside to mediate uptake and transcytosis across the respiratory epithelium^8^. In erythroid progenitor cells, globoside is dispensable for uptake but, upon endosomal acidification, it becomes essential for viral escape into the cytosol^9^. Thus, globoside acts as a conditional receptor supporting entry through the respiratory epithelium and endosomal escape in a strict pH-dependent manner.

B19V capsid is composed of VP1 and VP2. VP1 has an N-terminal extension of 227 amino acids, the so-called VP1 unique region (VP1u). Previous work demonstrated that B19V uptake is a highly selective process controlled by VP1u and restricted to permissive erythroid cells^10,11^. Subsequent mapping and mutational analyses localized a discrete receptor-binding domain (RBD) within the N-terminal region of VP1u that is necessary and sufficient to mediate erythroid-restricted internalization^12^. The unknown specific host receptor that is recognized by VP1u has been termed VP1uR^13^.

The functional properties of the VP1u RBD have been extensively characterized using heterologous display systems. Multivalent presentation of VP1u on different bacteriophage particles recapitulates the erythroid-restricted uptake profile of native B19V, competes with the virus for entry, and serves as a sensitive probe for receptor expression^11,13^. These studies established that VP1u-dependent uptake is confined to erythropoietin-dependent stages of erythroid differentiation and coincides precisely with the cellular window permissive for productive B19V infection^10,11,14^. Comparative analyses further revealed that VP1u-mediated uptake is conserved among primate erythroparvoviruses, supporting the existence of an evolutionarily conserved cellular receptor^13^. A recent study demonstrated that VP1u-dependent uptake also occurs in villous trophoblasts of the human placenta, showing that expression of a functional VP1uR extends viral entry competence beyond the erythroid lineage and providing a mechanistic basis for how B19V can traverse the placental barrier and reach the fetus^15^.

Despite this extensive functional characterization, the molecular identity of the cellular receptor mediating VP1u-dependent virus uptake has remained elusive. Identifying the VP1uR is essential for understanding the fundamental mechanism that governs B19V tropism and viral entry, both in the target erythroid progenitors and in transplacental transmission. To address this long-standing question, we investigated cell receptors engaged by the VP1u RBD in erythroid cells. Using a combination of proximity labeling, cell-based binding and uptake assays, direct binding experiments, and structural approaches, we have confirmed that human transferrin receptor 1 (TfR1/CD71) is recognized and binds specifically to the VP1u RBD. Furthermore, blocking TfR1 accessibility with the specific antibody OKT9 indicates that B19V interaction with TfR1 is essential for virus uptake.

## Results

### Proximity labeling identifies TfR1 as a VP1u-proximal cell surface protein

To identify host factors in close proximity to VP1u at the cell surface, we applied the enzyme-based Selective Proteomic Proximity Labeling Assay Using Tyramide (SPPLAT)^16^. In this approach, horseradish peroxidase (HRP) generates short-lived tyramide radicals in the presence of H₂O₂, resulting in covalent biotin labeling of proteins within a restricted spatial radius (Supplementary Fig. 1).

**Fig. 1:**
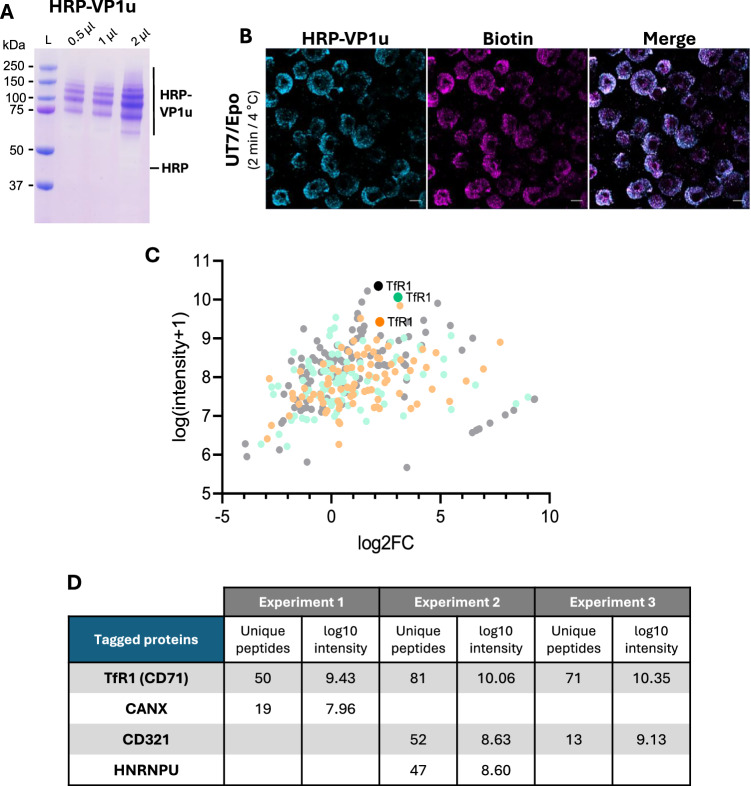
Proximity labeling identifies TfR1 as a VP1u-proximal cell surface protein. **A** SDS-PAGE analysis of maleimide-conjugated HRP-VP1u. L; ladder. **B** Proximity labeling in UT7/Epo cells. HRP-VP1u was bound to cells at 4 °C, followed by pulse labeling. HRP-VP1u and biotinylated proteins were detected using anti-FLAG and anti-biotin antibodies, respectively. A representative confocal image is shown. Scale bar: 10 μm. **C** Mass spectrometry analysis of affinity-purified biotinylated proteins. Proteins identified in three independent experiments were plotted according to log₂ fold change (log2FC) relative to HRP-only controls and protein intensity. Common hits are shown in distinct colors, with TfR1 highlighted. **D** Proteins identified as containing tyramide-modified tyrosine residues in three independent experiments. TfR1 is highlighted in red and represents the only protein detected in all three experiments. CANX Calnexin, CD321 F11 receptor, HNRNPU heterogeneous nuclear ribonucleoprotein. Source data are provided as a Source Data file. Experiments in panels A and B were independently repeated twice with similar results.

Recombinant VP1u was covalently conjugated to HRP, and the integrity of the conjugates was verified biochemically prior to use (Fig. 1A). UT7/Epo cells were incubated with HRP-VP1u at 4 °C to permit surface binding while preventing internalization. Cells exposed to unconjugated HRP and VP1u served as controls. Following activation of the labeling reaction with biotinylated tyramide, biotin deposition was assessed by immunofluorescence. HRP-VP1u and biotin signals showed strong colocalization at the plasma membrane, whereas control conditions yielded only background staining, confirming proximity-dependent labeling at the VP1u binding site (Fig. 1B).

Biotinylated proteins were affinity-purified and subjected to quantitative mass spectrometry. Peptides bearing tyramide-modified tyrosines were prioritized and filtered against the Cell Surface Protein Atlas (CSPA) database to restrict analysis to bona fide plasma membrane proteins^17^. Across three independent experiments, transferrin receptor 1 (TfR1/CD71) was the only reproducibly tyramide-labeled surface protein common to all datasets. Other candidates were either inconsistently detected between replicates or corresponded to proteins without well-established cell surface localization. Comparative analysis of shared proteins based on log₂ fold-change and absolute intensity consistently revealed TfR1 as highly enriched relative to controls (Fig. 1C, D). Together, these data identified TfR1 as the most robust and reproducible VP1u-proximal cell surface protein emerging from the proximity labeling screen.

### VP1u, but not B19V, colocalizes with TfR1 at the plasma membrane

To determine whether B19V directly engages TfR1 at the cell surface, we compared the binding pattern of recombinant VP1u with that of intact virions. Recombinant full-length VP1u containing a C-terminal cysteine residue to enable dimerization was expressed and purified as previously described^12^ and detected via its C-terminal FLAG tag. Cells were incubated with recombinant FLAG-tagged VP1u or with native B19V at 4 °C to permit surface binding while preventing endocytosis, thereby restricting the analysis to primary attachment events. TfR1 was visualized using an antibody directed against its cytosolic tail, avoiding potential interference with ligand binding at the ectodomain. Intact virions were detected using a monoclonal antibody specific for conformational epitopes of the capsid.

**Fig. 2:**
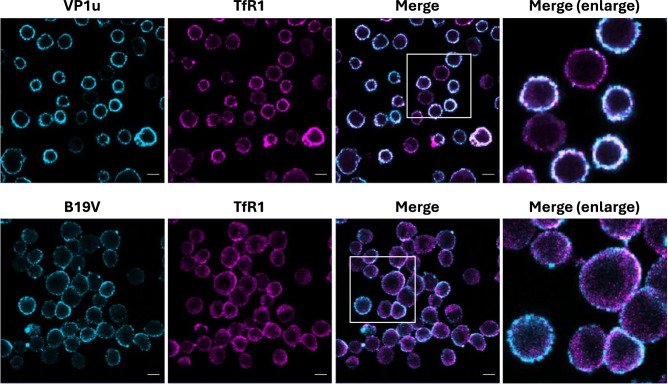
Recombinant VP1u colocalizes with TfR1 at the plasma membrane, whereas intact B19V does not. Confocal microscopy analysis of UT7/Epo cells incubated at 4 °C with recombinant FLAG-tagged VP1u or intact B19V to assess primary surface interactions. Top panels: VP1u (cyan) and TfR1 (magenta). Merged images demonstrate substantial colocalization at the cell periphery. Bottom panels: B19V (cyan) and TfR1 (magenta). Merged images show distinct spatial distribution with no evident colocalization. Enlarged views highlight the differential overlap patterns. TfR1 was detected using an antibody directed against its cytosolic tail. Recombinant VP1u was detected via its C-terminal FLAG tag, and native B19V was detected using a monoclonal antibody specific for intact capsids (mAb 860-55D). Scale bar: 10 μm. The experiment was independently repeated twice with similar results.

Confocal microscopy revealed pronounced colocalization of recombinant VP1u with TfR1 at the plasma membrane, as evidenced by strong signal overlap in merged images. In contrast, surface-bound B19V displayed a distinct distribution pattern with no evident colocalization with TfR1 under identical conditions (Fig. 2). These observations indicate that while the VP1u associates with TfR1 at the cell surface, intact virions do not measurably colocalize with the receptor during initial attachment.

### Antibody-mediated blockade of TfR1 differentially affects MS2-VP1u and B19V entry

To evaluate the role of TfR1 in VP1u-mediated B19V entry, we used the well-characterized murine monoclonal antibody OKT9, which recognizes the apical domain of human TfR1^18,19^. OKT9 has been widely used to inhibit TfR1-dependent viral entry, specifically for viruses that engage the apical domain of TfR1. We therefore used OKT9 to assess whether steric blockade of the TfR1 ectodomain affects VP1u binding and uptake, as well as B19V attachment, internalization, and infection.

**Fig. 3:**
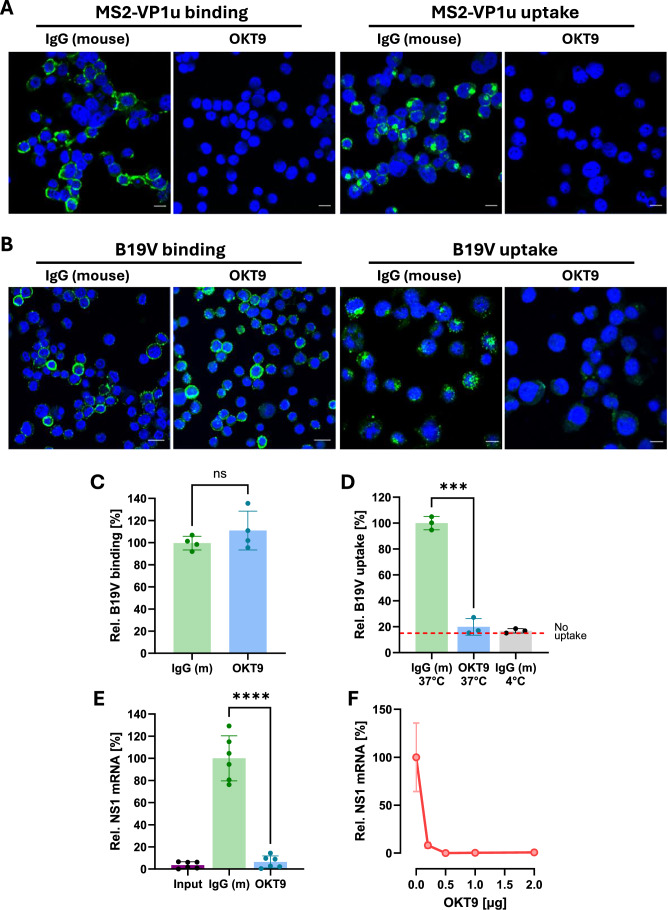
OKT9-mediated TfR1 blockade inhibits VP1u binding, B19V uptake, and infection. **A** Confocal microscopy of UT7/Epo cells preincubated with OKT9 or isotype-matched IgG control and exposed to MS2-VP1u-Atto 488 for 30 min at 4 °C (binding) or 37 °C (internalization). Scale bar: 10 μm. **B** Confocal microscopy of cells pretreated with OKT9 or IgG and incubated with B19V. B19V was detected using a monoclonal antibody specific to intact capsids (mAb 860-55D). Scale bar: 10 μm. **C** Quantification of B19V binding at 4 °C measured by qPCR. **D** Quantification of internalized B19V genomes following uptake at 37 °C. Trypsinization was used to remove non-internalized particles. **E** NS1 mRNA levels 24 h post-infection following preincubation with 2 µg OKT9. IgG (m); IgG (mouse). **F** Dose-dependent inhibition of NS1 mRNA synthesis following preincubation with increasing concentrations of OKT9 (0, 0.2, 0.5, 1, and 2 µg). All results are presented as the mean ± SD from biologically independent experiments (C, *n* = 4; D, *n* = 3; E, *n* = 6; F, *n* = 3). Statistical significance was calculated using an unpaired two-sided Welch’s test. Panel C, ns (*p* = 0.2933); panel D, *p* = 0.0001; panel **E**, p < 0.0001. Source data are provided as a Source Data file.

Preincubation of UT7/Epo cells with OKT9 completely abolished both binding and uptake of MS2 bacteriophage capsids covalently conjugated to VP1u (MS2-VP1u) via click chemistry, as visualized by confocal microscopy (Fig. 3A). In contrast, in cells treated with the same concentration of an isotype-matched mouse IgG control antibody, MS2-VP1u displayed strong surface binding and internalization. These data demonstrate that OKT9 binding to TfR1 prevents VP1u attachment to and uptake by host cells.

We next examined intact B19V under analogous conditions. Cells were preincubated with OKT9 for 30 min at 4 °C prior to virus addition. When binding was assessed at 4 °C, OKT9 did not reduce the intensity or distribution of surface-associated virus compared to the IgG control (Fig. 3B), suggesting that initial host cell attachment does not require an interaction with TfR1 apical domains. In contrast, when uptake was permitted at 37 °C for 30 min, OKT9 treatment blocked viral internalization as assessed by confocal microscopy (Fig. 3B). Quantitative PCR analysis of cell-associated and internalized viral genomes corroborated these observations. For this assay, non-internalized particles were removed by trypsinization prior to DNA extraction. The results confirmed that OKT9 blockade does not impair initial binding but prevents subsequent uptake (Fig. 3C, D).

Finally, the requirement for TfR1 interaction in B19V uptake and productive infection was assessed by preincubation of cells with 2 µg OKT9 prior to B19V infection, followed by quantification of NS1 mRNA levels 24 h post-infection. The OKT9 blockade completely inhibited NS1 expression compared with IgG-treated controls (Fig. 3E). Titration of OKT9 (0, 0.2, 0.5, 1, and 2 µg) revealed a dose-dependent inhibition of NS1 mRNA synthesis, reaching near-complete suppression at 0.2 µg (Fig. 3F), indicating that efficient B19V infection depends on TfR1 interaction. Together with the binding and uptake measurements, these data show that TfR1 is not required for initial virion attachment but must be engaged for subsequent internalization. These findings are consistent with a model in which B19V first associates with additional surface factor(s) before VP1u-dependent engagement of TfR1 triggers uptake and productive entry.

### VP1u binding is restricted to erythroid cells despite widespread TfR1 expression

Previous work has consistently demonstrated that VP1u binding and B19V uptake are restricted to erythroid cells, whereas non-erythroid cell types fail to support VP1u binding or viral uptake^14–16^. Following identification of TfR1 as the VP1u-binding receptor, we examined whether this restriction reflects differences in TfR1 surface expression or instead depends on erythroid-specific factors independent of overall receptor abundance.

**Fig. 4:**
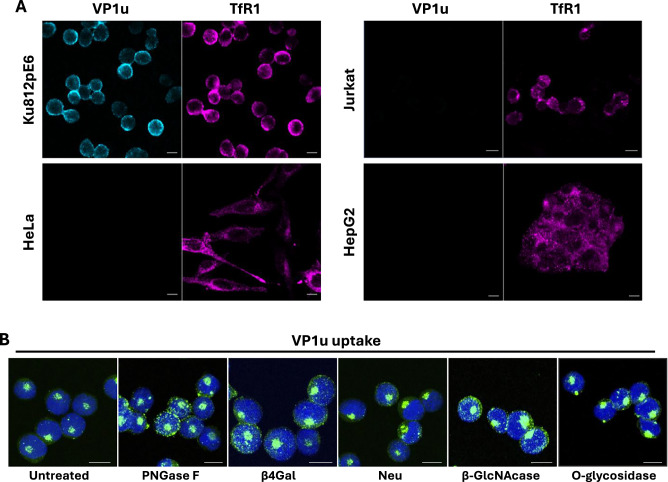
VP1u binding is restricted to erythroid cells and is independent of TfR1 glycosylation. **A** Confocal microscopy of erythroid (Ku812Ep6), and non-erythroid cells (HeLa, Jurkat and HepG2 cells) incubated with recombinant FLAG-tagged VP1u at 4 °C. VP1u was detected with anti-FLAG antibody (cyan) and TfR1 with an antibody against its cytosolic domain (magenta). **B** UT7/Epo cells treated with PNGase F, β1-4-galactosidase (β4Gal), neuraminidase (Neu), β-N-acetylglucosaminidase (β-GlcNAcase), or O-glycosidase prior to incubation with recombinant FLAG-tagged VP1u at 4 °C. VP1u binding was assessed by confocal microscopy. DAPI (blue). Scale bar: 10 μm. Experiments in panels (**A** and **B**) were independently repeated twice with similar results.

Non-erythroid cell lines representing distinct tissue origins, including HeLa (epithelial), HepG2 (hepatic), and Jurkat (T lymphoid) cells, were analyzed for TfR1 expression and VP1u binding. In parallel, Ku812Ep6 cells were included as an erythroid-like model. Ku812 cells, derived from a patient with chronic myelogenous leukemia^20^, display erythroid-associated features and can undergo erythroid differentiation in response to erythropoietin^21^. A highly permissive subclone, KU812Ep6, was generated by erythroid enrichment and limiting dilution and has been extensively used, together with UT7/Epo cells, as a model system to study B19V binding, uptake, and infection^22^. Cells were incubated with recombinant FLAG-tagged VP1u at 4 °C to assess surface binding, and TfR1 was detected using an antibody directed against its cytosolic tail following fixation and permeabilization. Confocal microscopy revealed a substantial plasma membrane-associated TfR1 expression in all cell types examined. However, VP1u binding was detected exclusively in KU812Ep6 cells and remained undetectable in non-erythroid cells under identical conditions (Fig. 4A).

These findings indicate that lack of VP1u binding in non-erythroid cells cannot be attributed to the absence of TfR1 at the cell surface and instead point to an erythroid-specific context required for productive VP1u-TfR1 interaction.

### TfR1 glycosylation does not affect VP1u uptake in erythroid cells

Because TfR1 is broadly expressed across diverse cell types whereas B19V selectively infects erythroid cells, we considered whether differences in receptor glycosylation might influence VP1u recognition. UT7/Epo cells were treated with glycosidases targeting defined carbohydrate moieties prior to incubation with recombinant FLAG-tagged VP1u. PNGase F was used to remove N-linked glycans, whereas β1-4-galactosidase, neuraminidase, and N-acetylglucosaminidase were used to trim terminal galactose, sialic acid, and N-acetylglucosamine residues, respectively. For O-glycosylation, samples were treated with O-glycosidase to remove core O-linked glycans. Confocal microscopy analysis showed that enzymatic removal of these carbohydrate structures did not reduce VP1u uptake (Fig. 4B).

Although the efficiency of enzymatic trimming was not independently quantified, the absence of any detectable reduction in VP1u uptake following treatment with multiple glycosidases indicates that accessible surface glycans are not essential determinants of the VP1u-TfR1 interaction in erythroid cells. These results therefore argue against differential TfR1 glycosylation as the basis for the erythroid restriction of VP1u binding and instead point to additional cell type-specific determinants beyond TfR1 glycan composition.

### VP1u binds to TfR1 in a cell-free system

To assess the VP1u-TfR1 interaction in a cell-free condition and determine the binding affinity between RBD and the receptor, we performed mass photometry and bio-layer interferometry (BLI) analysis. Monomeric and dimeric VP1u proteins were incubated with TfR1 (final concentration 3 µM) at a ratio of 2:1 and 1:1, respectively, for the mass photometry experiment, which confirmed the formation of the VP1u-TfR1 complex (Supplementary Fig. 2).

For the BLI analysis, Fc-tagged TfR1 was immobilized on Fc-capture biosensors, and the association and dissociation kinetics of VP1u monomer and dimer were measured using serial dilutions (Supplementary Fig. 3). The resulting graphs showed concentration-dependent binding kinetics; however, the data were not consistent with a 1:1 Langmuir binding model for both samples. Instead, the data were best fitted using a heterogeneous ligand (2:1) model, which yielded R^2^ values higher than 0.98, suggesting the presence of two different binding populations on the sensor surface. The calculated K_D_1 (K_D_2) values were about 3.1 (3.7) µM for the TfR1-VP1u monomer and 2.6 (4.5) µM for the TfR1-dimer, respectively (Supplementary Fig. 3). We did not observe significant avidity effects in the TfR1-VP1u dimer interaction, suggesting the dimer neither engaged dimeric TfR1 in a bivalent manner nor cross-linked two TfR1 molecules.

Consistent with these observations, experiments in UT7/Epo cells showed only a modest increase in surface binding of the VP1u dimer compared with the monomer when binding was measured at 4 °C. In contrast, assays performed at 37 °C, which allow receptor-mediated uptake, revealed a pronounced advantage of the VP1u dimer (Supplementary Fig. 4). These findings are in agreement with previous work demonstrating that VP1u dimers bind and internalize more efficiently than monomeric VP1u^12^.

Together, these results indicate that dimerization does not substantially increase the intrinsic affinity of VP1u for TfR1 but enhances functional interaction with the receptor in a cellular context. One possible explanation is that dimerization facilitates more efficient engagement of TfR1 at the cell surface, potentially involving local receptor clustering or cooperative interactions that can occur in the membrane environment. Such effects would not be captured in the BLI assay, where accessible TfR1 molecules are immobilized on the biosensor surface and lack lateral mobility.

### Cryo-EM 3D reconstruction reveals VP1u binding to TfR1 apical domain

To elucidate the molecular interaction between VP1u and TfR1 and determine the three-dimensional structure of the complex, we performed cryo-EM single-particle analysis. Because monomeric VP1u interacts with dimeric TfR1, we reasoned that two VP1u monomers would bind to each TfR1 dimer. Monomeric VP1u was therefore used to avoid cross-linking of TfR1 through bivalent binding by VP1u dimers. Full-length VP1u was incubated with the recombinant human TfR1 ectodomain at a 2:1 molar ratio, and the resulting complex was vitrified for cryo-EM data collection and single-particle reconstruction (Supplementary Table 1).

**Fig. 5:**
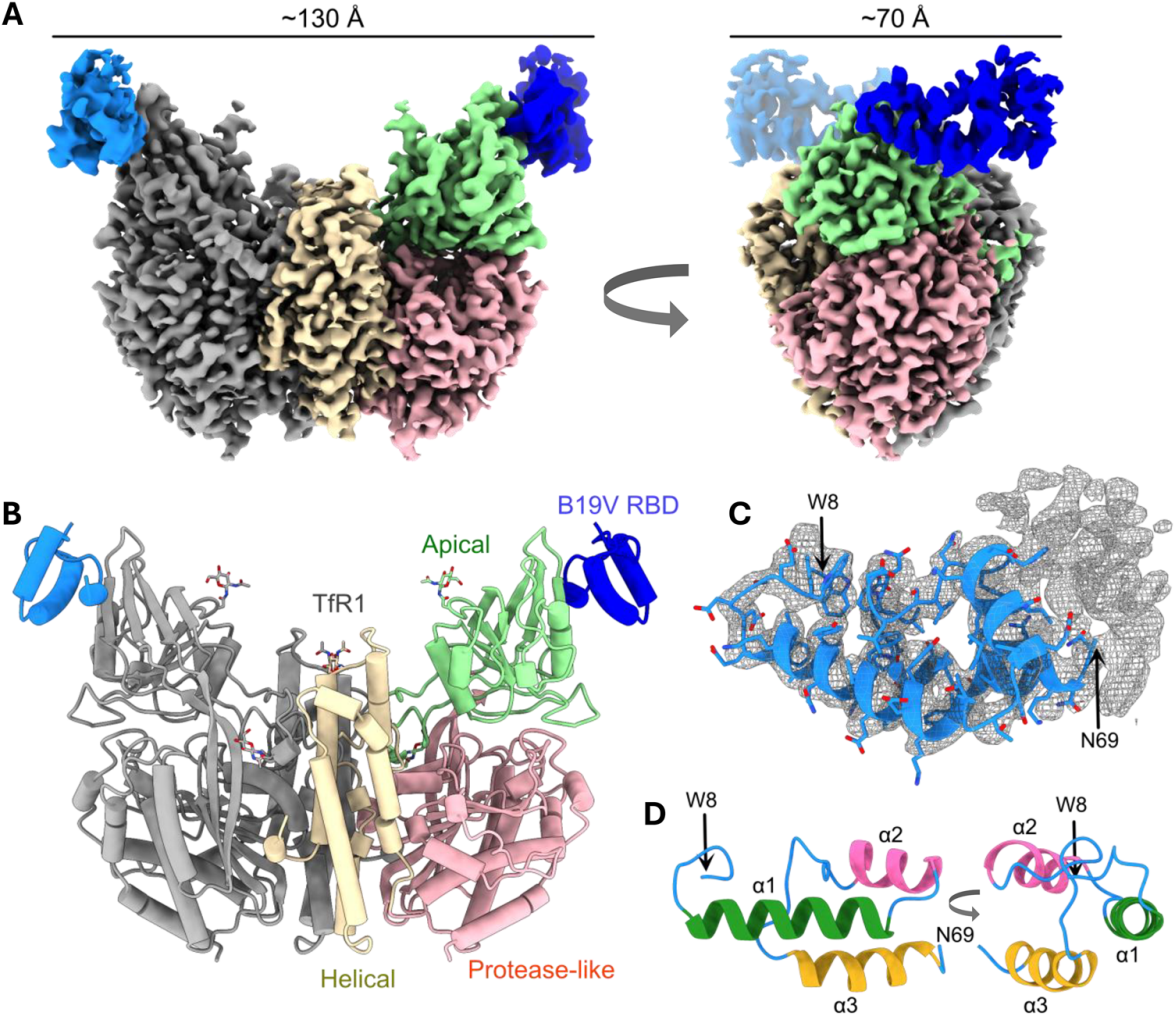
VP1u RBD binds to the apical domain of TfR1. **A** Cryo-EM 3D reconstruction of TfR1 in complex with recombinant VP1u monomer. One TfR1 monomer is colored to distinguish the apical, protease-like, and helical domains. The two additional densities corresponding to the two bound VP1u molecules are shown in blue and light blue. **B** Atomic model of the TfR1-VP1u RBD complex structure, depicted as a ribbon diagram with cylinders and stubs representing α-helices and β-strands, respectively. **C** De novo model of RBD (residues 8-69) with cryo-EM density, shown as a mesh envelope. **D** The atomic model of the VP1u RBD displayed as a ribbon diagram. The three α-helices are highlighted and labeled.

The three-dimensional map of the VP1u-TfR1 complex was initially reconstructed at 2.23 Å resolution using 681,957 particles with *C*_2_ symmetry (Supplementary Fig. 5 and 6A). In parallel, the TfR1 ectodomain alone was vitrified and analyzed, yielding a structure at 2.43 Å resolution (Supplementary Fig. 6B and 7). Both maps revealed the canonical TfR1 architecture, characterized by a butterfly-shaped dimeric ectodomain composed of apical, protease-like, and helical domains. Notably, in the VP1u-TfR1 complex map, two additional unfilled densities were observed, one on the side of each apical domain, which were of dimensions that could correspond to the receptor-binding domains (RBDs) of VP1u (Supplementary Fig. 5 and Fig. 5A).

Although the TfR1 density was well defined, the additional densities exhibited weaker signal intensity and lower local resolution, particularly in their distal regions (Supplementary Fig. 8), suggesting partial occupancy and structural flexibility. To improve the putative RBD density, particles were symmetry-expanded and subjected to two rounds of focused classification for each RBD, removing approximately 55 % of particles with partial occupancy (Supplementary Fig. 5). The reconstruction from the selected particle images showed enhanced RBD density, presumably reflecting full occupancy, albeit at a slightly lower overall resolution of 2.41 Å (Supplementary Fig. 6C). Furthermore, the classified symmetry-expanded particles were used for three-dimensional flexible reconstruction, which substantially improved the overall quality and continuity of the VP1u density (Supplementary Fig. 5 and Fig. 5A).

### Atomic model building identifies VP1u RBD

We first refined the atomic model of TfR1 against the cryo-EM density map using the crystal structure of human TfR1 as the initial model (Supplementary Figs. 6D and 9)^23^. Comparison of the atomic models of the cryo-EM and crystal structures of TfR1 revealed conformational differences in the apical traverse loop, which spans the interface between the apical and protease-like domains, and in the C-terminal tail of the polypeptide chain, with local Cα RMSDs of 5.543 Å and 4.630 Å, respectively (overall Cα RMSD: 1.669 Å) (Supplementary Fig. 9B–D). Both regions coordinate Sm^3+^ ions in the crystal structure^23^, likely reflecting variations in experimental conditions. The TfR1 model was then refined against the VP1u-TfR1 complex density map (Fig. 5B), revealing local conformational change in the apical loop that engages VP1u when compared with the TfR1-alone structure (overall Cα RMSD: 0.664 Å) (Supplementary Fig. 9E, F).

After finalizing the TfR1 model, the remaining unassigned density was used for de novo model building with ModelAngelo^24^, guided by the amino acid sequences of the VP1u RBD (residues 1-71). We also tested using the full-length sequence (residues 1-227). Both sequences generated models with identical amino acid registration, which were further refined to produce an atomic model corresponding to residues 8-69 that was built into the density with high confidence (Fig. 5C). Consistent with previous predictions^12^, the VP1u receptor-binding domain contains three α-helices (α1– α3) that assemble into a compact three-helix bundle fold (Fig. 5D). No density was observed N-terminal to Trp8, whereas weak and disordered density extended from Asn69 (Fig. 5C). The remaining C-terminal region of VP1u, including the phospholipase A2 (PLA2) domain, was not resolved in the map. An Alphafold 3 model of VP1u predicts structures for both the RBD and the PLA2 domains (Supplementary Fig. 10)^25^. However, Alphafold 3 indicated a low confidence value (pLDDT <50) for residues 79-121 between the RBD and PLA2 domains (Supplementary Fig. 10), suggesting that this region is flexible or intrinsically disordered. Thus, although RBD binding to the TfR1 apical domain is resolved, the adjacent long flexible polypeptide chain likely hinders reconstruction of the downstream VP1u region, including the PLA2 domain. It is possible that the unresolved C-terminal region influenced the binding of another VP1u on the opposite side of the TfR1, which may explain the non-Langmuir binding behavior we observed in BLI analysis (Supplementary Fig. 3).

Three glycosylation sites exhibited clear density corresponding to the first glycan residues at Asn251, Asn317, and Asn727 (Fig. 5B), although more extended glycan density was observed only at lower, noisier contour levels (Supplementary Fig. 5 and 7). None of these glycosylation sites were positioned close enough to interact with the VP1u receptor-binding domain. We also analyzed the VP1u-TfR1 complex structure using PRODIGY web server^26^ to predict the binding affinity between RBD and TfR1, yielding a K_D_ of 1 μM, which is within the same order of magnitude as the BLI measurements. Overall, the cryo-EM structure of the VP1u-TfR1 complex demonstrated that two VP1u RBDs bind, one to each TfR1 apical domain on each side of the TfR1.

### Molecular interactions between RBD and TfR1

**Fig. 6:**
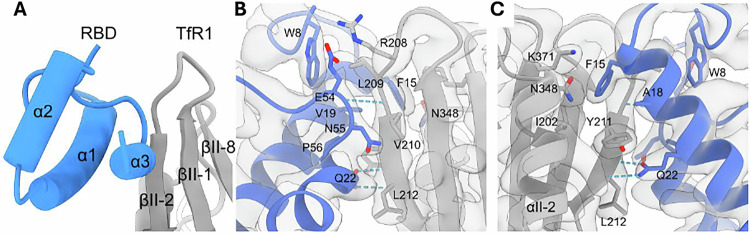
Molecular interaction between VP1u RBD and TfR1. **A** Close-up view of the RBD-TfR1 binding interface from Fig. 5C. Secondary structure elements of the RBD and TfR1 are labeled. **B**,** C** Contacting residues between RBD and TfR1 are shown as stick representations with side chains displayed and residues labeled. The cryo-EM map of the TfR1-VP1u complex is displayed as a transparent isosurface.

Using the atomic model of the VP1u-TfR1 complex, we identified the contact residues between the RBD and the receptor. The N-terminal region, α2-α3 loop, and the α1 and α3 helices of the RBD interacted with the apical domain of TfR1 (Fig. 6A), primarily through the βII-1-βII-2 loop and the βII-2 strand (residues 208-212) (Fig. 6B), as well as three residues from the βII-1 strand, βII-8 strand, and αII-2 helix (I202, K371, and N348, respectively) (Fig. 6C). Notably, Phe15 of the RBD is the only residue that contacts all three residues outside the βII-2 strand, whereas the remaining RBD residues interact exclusively with the βII-1-βII-2 loop and the βII-2 strand (Fig. 6C).

**Fig. 7:**
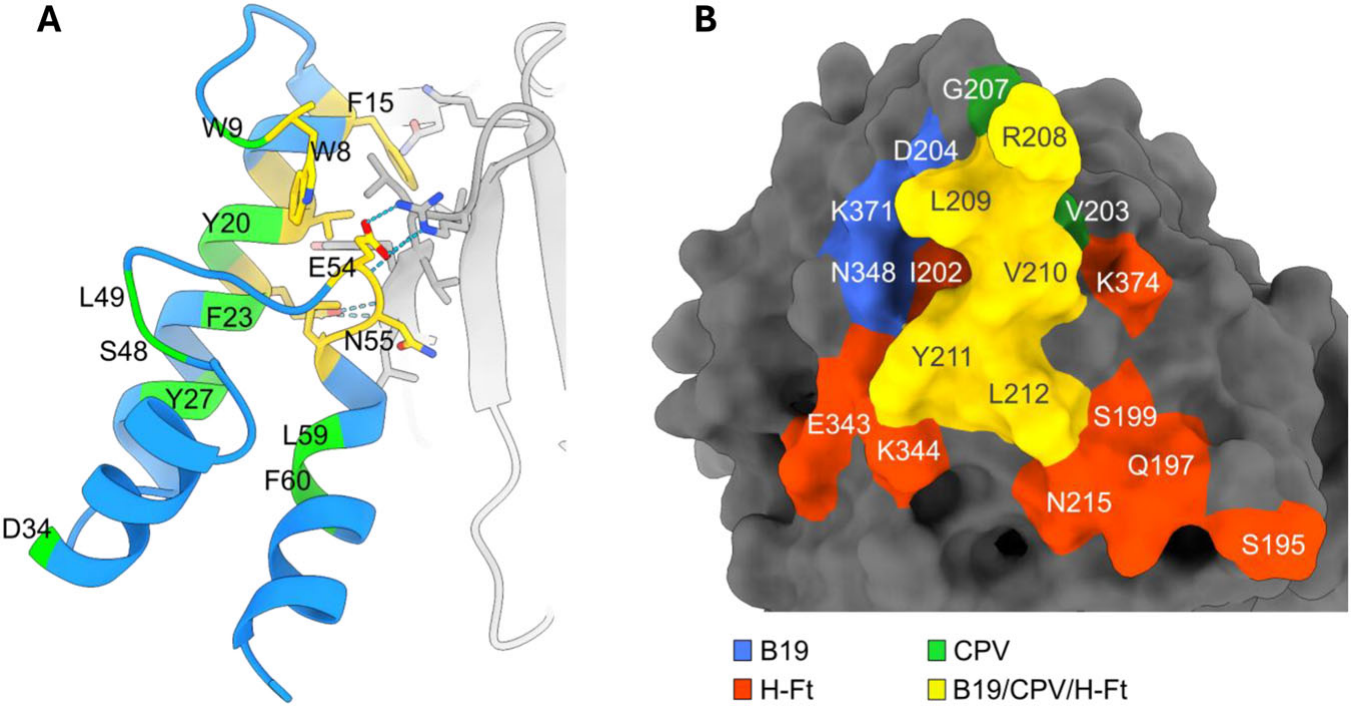
Mapping of critical residues. **A** Residues previously examined for their functional roles in VP1u internalization are highlighted. Residues located within the contact interface with TfR1 are colored yellow, whereas those not found at the interface are colored green. **B** Binding sites of the B19V RBD, human ferritin, and canine parvovirus (CPV) are mapped on the surface of human TfR1. Each interaction site is colored according to the key shown at the bottom. Residue numbering of canine TfR1 was aligned with that of human TfR1. Only consistent contacts for CPV are shown^28^.

Previously, we identified a cluster of amino acids that play critical roles in VP1u internalization through mutational analysis^12^. Mapping these functionally important residues onto the RBD-TfR1 structure revealed that many residues, including WW8/9, F15, V19, Q22, and EN54/55, are located at the receptor-binding interface, whereas the remaining residues are positioned at the helical interface, where mutations are likely to affect RBD stability (Fig. 7A). Notably, most of the contact residues identified in the RBD-TfR1 complex structure, except for one untested residue (Pro 56) (Fig. 6B), exhibited significantly reduced VP1u activity when mutated^12^, supporting the conclusion that the previously unidentified VP1u receptor (VP1uR) in our earlier study was in fact TfR1.

We then compared the RBD contact residues with those involved in interactions with human ferritin and canine parvovirus (Fig. 7B). All three molecules commonly engaged residues 208-212 on the βII-1-βII-2 loop and the βII-2 strand. The human ferritin and CPV binding sites involve broader surface areas, likely reflecting the larger, spherical shapes of these molecules, although CPV-TfR1 contacts are transient^27^. In contrast, the interaction interface between the VP1u RBD and TfR1 covers a smaller area of about 480 Å², consistent with the relatively weak binding affinity. The residues on the βII-1 and βII-8 strands and the αII-2 helix (I202, K371, and N348) are unique to B19V contacts. Residue K371 is also found among the contact residues of other human pathogens, including Machupo virus (MACV)^28^ and the parasite Plasmodium vivax^29^.

## Discussion

B19V is characterized by a remarkably strict tropism for EPCs, a restriction that underlies its pathogenesis^30^. Extensive work over the past decade established that this narrow tropism is primarily controlled at the level of viral uptake and is mediated by VP1u, specifically via an RBD located within its N-terminus^10,13,14^. VP1u RBD is necessary and sufficient to drive erythroid-restricted internalization, and its uptake profile precisely matches the differentiation window permissive for productive infection^11^. Despite this detailed functional characterization, the molecular identity of the VP1u receptor has remained unresolved. AXL was recently proposed to contribute to B19V entry into EPCs. However, AXL expression is not restricted to erythroid cells, and inhibition or ectopic expression experiments indicate that AXL alone is not sufficient to confer full susceptibility to B19V infection. These observations suggest that AXL may act as a contributory entry factor rather than the primary receptor responsible for VP1u-mediated uptake^31^.

Here, we identify TfR1 (CD71) as a specific binding partner of VP1u and show that it corresponds to the previously postulated VP1u receptor (VP1uR) required for VP1u-mediated viral internalization. Proximity labeling reproducibly identified TfR1 as the only consistently enriched cell surface protein across independent experiments. Recombinant VP1u colocalized with TfR1 at the plasma membrane under conditions restricting analysis to primary binding events, whereas intact virions did not show detectable colocalization at this stage. Functional blockade of the TfR1 apical domain with OKT9 abolished VP1u binding and uptake and prevented B19V internalization and infection, while virus attachment at 4 °C was not affected. The inhibitory effect was dose-dependent and nearly complete at low antibody concentrations, further supporting the functional relevance of this interaction. In parallel, cell-free binding assays confirmed the VP1u-TfR1 binding and measured the binding kinetics. Cryo-EM analysis demonstrates direct binding of the VP1u RBD to the TfR1 ectodomain and defines the molecular interface at near-atomic resolution. Residues previously shown by mutagenesis to be critical for VP1u uptake localize directly to the receptor-binding surface in the structure, providing independent validation of the identified interface^12^. The convergence of proteomic enrichment, antibody blockade, uptake assays, infection readouts, biochemical assays, and structural characterization provides orthogonal evidence that TfR1 is the receptor engaged by VP1u to trigger B19V internalization.

The differential effect of TfR1 blockade on attachment versus internalization supports a multistep mechanism for B19V entry. The observation that virion attachment occurs independently of TfR1, whereas internalization requires TfR1 engagement, indicates that intact B19V first associates with the erythroid cell surface through VP1u-independent interaction(s) before engaging TfR1. This sequential mechanism is consistent with our previous demonstration that VP1u is not exposed on the native capsid surface but becomes accessible only after contact with permissive erythroid cells^32^. More recently, we showed that patient-derived B19V particles circulate coated with host-derived protease inhibitors that dissociate upon interaction with erythroid cells, accompanied by structural rearrangements and exposure of the otherwise concealed VP1u region^33^. These findings support a priming-dependent entry mechanism in which native virions initially attach to the host through capsid-exposed determinant(s) independent of TfR1, followed by conformational remodeling that enables VP1u engagement of TfR1, which subsequently triggers internalization. The identity of the primary attachment factor remains to be defined, but the present data separate the attachment step from the TfR1-dependent uptake step.

A second central question concerns the restriction of VP1u-TfR1 interaction to erythroid cells despite widespread TfR1 expression. The present data impose important constraints. In previous studies and in this study, multiple non-erythroid cell lines express TfR1 yet do not support VP1u binding^10,13^. Enzymatic removal of N-linked glycans and terminal carbohydrate residues from UT7/Epo cells does not impair VP1u binding. Furthermore, the cryo-EM structure demonstrates direct binding of VP1u to the recombinant TfR1 ectodomain produced in HEK293 cells, indicating that non-erythroid-derived TfR1 is intrinsically capable of interaction. This apparent discrepancy indicates that the inability of VP1u to bind non-erythroid cells does not reflect an intrinsic incompatibility of the receptor but instead arises from the cellular context in which TfR1 is presented at the cell surface. Together, these observations argue against models based on erythroid-specific TfR1 isoforms or glycosylation-dependent recognition.

Many viral pathogens use TfR1 for their entry, including Japanese encephalitis virus^34^, SARS-CoV-2^35^, as well as influenza virus^36^. Our structural mapping reveals that B19V specifically targets the apical domain of TfR1. This region is also engaged by unrelated pathogens, including influenza virus^36^, New World arenaviruses^19^, and carnivore parvoviruses^27,37,38^, defining the importance of the apical surface. Consistent with this trend, the apical domain of TfR1 has been identified as an evolutionary hotspot shaped by host-virus arms races^39^. In another parvovirus, AAV, engineering the AAV9 capsid to bind the human TfR1 apical domain enabled the vector to actively cross the blood-brain barrier and achieve efficient, brain-wide gene delivery^40^. In contrast to carnivore parvoviruses and AAV, B19V mediates receptor binding through a specialized capsid extension (VP1u) rather than the icosahedral capsid topology^27^. The VP1u-TfR1 structure therefore illustrates how a modular capsid element can govern cell-type-restricted uptake while the structural shell remains functionally distinct.

Structural considerations indicate that simultaneous engagement of both apical domains of a TfR1 dimer by VP1u is geometrically feasible from a single virion. The spacing between TfR1 binding sites (~90 Å) is comparable to the distance between neighboring fivefold axes on the B19V capsid (~120–130 Å), and the length of VP1u is sufficient to span this range. Accordingly, two VP1u molecules from one or adjacent fivefolds could, in principle, engage a TfR1 dimer. Consistent with this, dimeric VP1u shows enhanced attachment to cells, indicating improved receptor engagement. However, this effect is not observed in BLI measurements, suggesting that bivalent engagement does not substantially increase intrinsic binding affinity. This likely reflects differences between binding to purified receptor in vitro and binding at the cell surface, where receptor organization or cooperative effects may influence engagement.

In summary, we have established VP1u-specific recognition and binding of TfR1. Furthermore, combined proteomic, functional, and structural evidence indicates that TfR1 is the previously unknown VP1u receptor (VP1uR) that is required for VP1u-dependent internalization of B19V. These data resolve a long-standing question in B19V biology by identifying the molecular receptor that mediates erythroid-restricted uptake and by defining its structural interface. The clear separation between primary attachment and TfR1-dependent internalization reveals the sequential organization of viral entry. Together, these findings provide a mechanistic framework for B19V uptake and establish TfR1 as the receptor mediating VP1u-dependent uptake into target erythroid progenitor cells.

## Methods

### Cells and viruses

UT7/Epo cells, provided by E. Morita (Tohoku University School of Medicine, Japan) were cultured in Eagle’s Minimum Essential Medium (MEM, Thermo Fisher Scientific, Waltham, MA, USA) with 5% FCS and 2 U/mL Epo. KU812Ep6 and Jurkat cells were maintained in RPMI 1640 with 10% FCS; KU812Ep6 cultures were additionally supplemented with 6 U/mL Epo. The hepatocarcinoma cell line HepG2 and HeLa cells were cultured in DMEM with 10% FCS. All cells were routinely maintained at 37 °C and 5% CO_2_.

Native B19V was obtained from de-identified plasma samples of infected, seronegative individuals, confirmed by virus-specific serology (CSL Behring, Bern, Switzerland). Infected plasma was thawed and clarified by centrifugation at 1800 × *g* for 10 minutes at 4 °C. To generate IgG-depleted B19V, the plasma was subjected to 20% (w/v) sucrose/PBS ultracentrifugation using a TLA100.3 rotor (Beckman) at 195,000 × *g* for 2 h at 4 °C. The viral pellet was resuspended in PBS and contaminant human IgG was removed by incubation with Protein G Plus Agarose (sc-2002, Santa Cruz Biotechnology) for 1 h at 4 °C. Viral genomes were quantified by quantitative PCR (qPCR) using Luna Universal One-Step Reaction Mix (M3003, New England Biolabs, NEB, Ipswich, MA, USA) with primers specific to the NS1-coding region of the genome as mentioned below. Plasmids containing the complete B19V genome were used as external standards in 10-fold serial dilutions.

### Recombinant VP1u constructs and MS2-VP1u VLPs

Recombinant full-length VP1u was expressed and purified as previously described^12,14^. Briefly, *E. coli* BL21(DE3) cells were transformed with pT7-FLAG-MAT-Tag-2 plasmids encoding either full-length VP1u or a modified VP1u containing an additional C-terminal cysteine residue to allow disulfide-mediated dimerization. The resulting proteins therefore yielded either monomeric VP1u or dimerizable VP1u variants, enabling comparison of monomeric and dimeric forms in subsequent experiments. Expression and purification of recombinant VP1u proteins were performed as previously described^14^.

For proximity labeling studies, a pT7-FLAG-MAT-Tag-2 vector encoding a C-terminally truncated VP1u of B19V (amino acids 1-99) was transformed into *E. coli* BL21(DE3) cells. This construct lacks the C-terminal 128 amino acids of the full-length VP1u (227 aa) while retaining a fully functional RBD^12^. A C-terminal cysteine residue was introduced to allow disulfide-mediated dimerization of the protein. Expression and purification of truncated VP1u proteins were performed as previously described^14^. Recombinant VP1u was conjugated to horseradish peroxidase (HRP). The recombinant VP1u was reduced by addition of 5 mM TCEP (Lucerna-Chem, P1021) for 30 min followed by addition of HRP-Maleimide (ImmunoChemistry Technologies, 6294), keeping the VP1u at a 30-fold molar excess. After 16 h at 4 °C, the reactants were separated using an ÄKTA size exclusion column (Superdex 200 increase 10/300) and fractions of 400 µl were collected. Collected proteins were separated by SDS-PAGE and the fractions containing the desired construct were pooled and subsequently concentrated using spin columns with a 30 kDa molecular weight cut-off (Merck Millipore, UFC503008). Concentrated HRP-VP1u was stored at −80 °C.

MS2-bacteriophage virus-like particles (VLPs) were expressed using *E. coli* BL21(DE3), and purified by ultracentrifugation through a 20% sucrose cushion at 150,000 × *g* for 4 h at 4 °C^13^. MS2-VLPs were purified and labeled with NHS-Atto 488 (Atto-Tec, Siegen, Germany) using a 40-fold molar excess of dye. The reaction was quenched, and labeled VLPs were recovered by ultracentrifugation through a 20% sucrose cushion at 150,000 × *g* for 4 h at 4 °C to remove unreacted dye. Subsequently, MS2 VLPs were incubated with 500-fold molar excess of maleimide-PEG_24_-NHS (22114, Thermo Fisher Scientific) for 1 h. Excess crosslinker was eliminated using a 40 kDa MWCO desalting column. The activated VLPs were then conjugated to reduced recombinant full-length VP1u proteins and further purified by a second 20% sucrose cushion.

### Proximity-based biotinylation of VP1u-proximal proteins

All the steps were performed at 4 °C unless stated otherwise. For each experiment 1.5 *× *10^7^ cells were washed twice with PBS at room temperature and then resuspended in PBS to reach a final concentration of 5 *× *10^6^ cells per ml. HRP-VP1u (5 µg/ml) was added and allowed to interact with the cells for 2 h under constant agitation. In parallel, a control sample was incubated with 4 µg/ml VP1u and 1 µg/ml HRP (unconjugated). Cells were pelleted at 700 *× g* for 5 min, washed once with ice-cold PBSA 0.2 % (PBS containing 0.2 % albumin), then resuspended in freshly prepared labeling buffer (50 mM Tris-HCl pH 7.4, 0.03 % H_2_O_2_, 80 µg/ml Tyramide-Biotin [Iris Biotech, LS-3570.0250, 10 mg/ml in DMSO]) and incubated for 2 min. Catalase (Sigma-Aldrich, C9322) was added to a final concentration of 100 U/ml to stop the reaction. Cells were then washed three times with ice-cold PBSA 0.2 %, followed by lysis using ice-cold lysis buffer 20 mM Tris-HCl pH 7.4, 5 mM EDTA pH 8.0, 150 mM NaCl, 1 % Triton X-100, 0.1 M sodium thiocyanate, 1× Protease inhibitor cocktail (500 µl per 5 *×* 10^6^ cells; 11836153001, Roche Diagnostics GmbH, Mannheim, Germany). Samples were vortexed briefly and incubated for 15 min before addition of Benzonase (Millipore, E1014) and incubation for 30 min. Samples were spun at 12,000 *× g* for 10 min to remove insoluble material. In the meantime, neutravidin beads (Thermo Fisher Scientific, 29200) were washed once with PBSA 1% and twice with lysis buffer. The lysate supernatant was added to the beads and incubated for 1 h under constant agitation. The beads were then washed four times with wash buffer 1 (10 mM Tris-HCl pH 7.4, 1 % Triton X-100, 1 mM EDTA pH 8.0, 0.5 % SDS, 500 mM NaCl, 0.1 M sodium thiocyanate, 1× Protease inhibitor cocktail) and twice with wash buffer 2 (10 mM Tris-HCl pH 7.4, 1 % Triton X-100, 1 mM EDTA pH 8.0, 0.5 % SDS, 0.1 M sodium thiocyanate, 1× Protease inhibitor cocktail). Beads were subsequently resuspended in 4X LDS sample buffer (Thermo Fisher, Waltham, MA).

### Identification of VP1u-proximal proteins

Purified biotinylated proteins were separated on an SDS-PAGE. After the gel front had migrated roughly 1.5 cm, the run was stopped, and proteins were stained using Coomassie gel stain (Thermo Fisher Scientific, 24615). A sterile scalpel was used to cut the resolved proteins of each lane into three horizontal slices. The slices were collected and stored in separate 1.5 ml tubes. Gel pieces were overlaid with 80 % ethanol and stored at 4 °C. Proteins were identified by MS analysis at the Core Facility Proteomics & Mass Spectrometry in Bern. The final analysis included three biologically independent SPPLAT experiments. Each experiment contained one HRP-VP1u sample and one control sample consisting of unconjugated HRP plus VP1u. Each sample was analyzed by two technical LC-MS/MS injections. Proteins were identified using MaxQuant version 2.0.1.0. Searches were performed against the UniProt Swiss-Prot human database supplemented with VP1/HRP sequences and common contaminants. Proteins identified by a single peptide were excluded from downstream analysis. Detailed LC-MS/MS acquisition parameters, raw data, and processed files are available through the PRIDE repository under accession number PXD076389. Quantification was performed using the sum of the three most intense peptide intensities (Top3). Variance Stabilizing Normalization (VSN) was employed before summing the peptides for Top3. Protein eEnrichment was calculated as log_2_ fold-change (log2FC) compared to the control. Additionally, peptides with tyramide-modified tyrosines were identified.

### Binding and internalization assays

Cell surface binding of recombinant VP1u (100 ng), MS2-VP1u particles and B19V (10⁵/cell) was carried out by incubating cells in PBS (pH 7.2) at 4 °C for 30 min. After incubation, cells were washed three times with ice-cold PBS and either subjected to DNA isolation followed by qPCR analysis or fixed for immunofluorescence microscopy. For internalization assays, MS2-VLPs, or B19V were incubated at 37 °C for 30 min. Cells were then washed with PBS. For qPCR-based analysis, cells were subsequently treated with trypsin/EDTA at 37 °C for 4 min to remove surface-associated particles, washed again, and processed for DNA extraction. For immunofluorescence analysis, cells were washed after internalization and processed directly for staining without trypsin treatment. Genomic DNA was purified using the GenCatch Plus Genomic DNA Miniprep Kit (1660250, Epoch Life Science, Missouri City, TX, USA) following the manufacturer’s protocol. Quantitative PCR was performed using the primers B19-F 5′-GGGGCAGCATGTGTTAAG-3′ and B19-R 5′- CCATGCCATATACTGGAACAC-3′.

### Antibody-mediated blockade of the VP1u-TfR1 interaction using OKT9

UT7/Epo cells were preincubated with 2 µg, unless otherwise indicated, of anti-human TfR1 monoclonal antibody OKT9 (103101-0 N; Caprico Biotechnologies, Duluth, MN) or with an isotype-matched mouse IgG control (14-4714-82; Thermo Fisher) for 30 min at 4 °C prior to addition of MS2-VP1u or B19V. For binding analyses, antibody-treated cells were incubated with MS2-VP1u or B19V at 4 °C for 30 min, washed extensively with cold PBS, and either fixed for confocal microscopy or processed for DNA extraction and qPCR. For uptake experiments, cells were incubated at 37 °C for 30 min to allow internalization. For samples analyzed by qPCR, surface-bound particles were removed by trypsin/EDTA treatment (4 min, 37 °C), followed by washing prior to DNA extraction. For samples analyzed by immunofluorescence, cells were washed after internalization and fixed directly without trypsin treatment. To assess productive infection, UT7/Epo cells were preincubated with OKT9 (0–2 µg) before infection with B19V. Total RNA was isolated 24 h post-infection, and NS1 mRNA levels were determined by qRT-PCR using the following primers: NS1-F: 5′-GGGGCAGCATGTGTTAAG-3′, B19-NS1-R: 5′- CCATGCCATATACTGGAACAC-3′.

### Immunofluorescence microscopy

Colocalization of VP1u with TfR1 was assessed in UT7/Epo cells by incubating recombinant B19 VP1u at 4 °C for 30 min in the presence of a monoclonal rabbit anti-FLAG IgG antibody (14793S, Cell Signaling Technologies, Danvers, MA, USA). The cells were washed several times with ice-cold PBS before fixation in a 1:1 mixture of methanol:acetone at −20 °C for 4 min. The FLAG antibody was detected with a polyclonal Alexa Fluor® 488 conjugated goat anti-rabbit IgG (Thermo Fisher). TfR1 was detected with a mouse anti-human TfR1 (13-6800; Thermo Fisher) specific to residues 3-28 of the human TfR1 cytoplasmic tail and a secondary polyclonal goat anti-mouse IgG conjugated to Alexa Fluor® 594 (Invitrogen). B19V capsids were detected using a monoclonal human IgG anti-B19 VP2 antibody (860-55D, Mikrogen, Neuried, Germany) and a secondary polyclonal goat anti-human IgG conjugated to Alexa Fluor® 488 (Invitrogen). All antibodies were diluted in 2% milk/PBS and incubated at 4 °C, after which specimens were treated with 2 mM CuSO_4_/50 mM NH_4_. The samples were mounted with EMS shield mount (17985-09, Electron MicroscopTy Sciences, Hatfield, PA, USA) supplemented with 0.2 ng/mL DAPI when indicated. The samples were visualized with a 63× oil immersion objective by laser scanning confocal microscopy (LSM880, Zeiss, Oberkochen, Germany).

### Enzymatic removal of cell surface glycans and VP1u binding assay

To evaluate whether glycosylation of TfR1 contributes to VP1u binding, UT7/Epo cells were subjected to enzymatic removal of specific surface glycans prior to incubation with recombinant VP1u. A total of 3 × 10⁵ cells per condition were washed once with PBS and resuspended in 30 μl of the corresponding reaction buffer. Cells were incubated for 1 h at 37 °C with one of the following enzymes under buffer-matched conditions: PNGase F (500 U; 50 mM sodium phosphate, pH 7.5; P0704, NEB), O-glycosidase (10 U; 50 mM sodium phosphate, pH 7.5; P0733, NEB), β1-4-galactosidase (10 U; 50 mM sodium acetate, pH 5.5, 5 mM CaCl₂; P0745, NEB), β-N-acetylglucosaminidase (10 U; 50 mM sodium acetate, pH 5.5, 5 mM CaCl₂; P0744, NEB), or α2-3,6,8 neuraminidase (40 U; 50 mM sodium acetate, pH 5.5; P0720, NEB). For removal of O-linked glycans, cells were first treated with neuraminidase, washed, and subsequently incubated with O-glycosidase (10 U; 50 mM sodium phosphate, pH 7.5; P0733, NEB). Following enzymatic treatment, cells were washed with PBS and incubated with recombinant FLAG-tagged VP1u at 37 °C for 1 h and processed for immunofluorescence analysis as described above.

### Mass photometry and Bio-layer interferometry

The purified VP1u proteins were incubated with tag-free TfR1 (Acro Biosystems) at room temperature for 30 min, at a final concentration of 4 µM TfR1 and 8 µM VP1u monomer or 4 µM VP1u dimer. The mass of the incubations was measured by using TwoMP (Refeyn). Molecular weights were calibrated using MassFerence P1 calibrant (Refeyn).

For BLI analysis, anti-hIgG Fc capture (AHC) biosensors (Sartorius) were used to bind Fc-tagged TfR1 (Acro Biosystems). Binding kinetics and affinity were measured using Octet R2 (Sartorius). The AHC biosensors were first blocked and hydrated in kinetics buffer (PBS with 0.01 % ovalbumin and 0.02 % Tween 20), which was used during the whole experiment. Binding experiments were performed with the following protocol: 60 s equilibration, 300 s of loading 10 μg/mL of TfR1, 180 s of baseline, 300 s of association with VP1u molecules, and 300 s of dissociation. The data were analyzed using Octet Analysis Studio with a 2:1 (heterogeneous ligand) binding model.

### Sample preparation and Cryo-EM data collection

The purified VP1u proteins were incubated with tag-free TfR1 (Acro Biosystems) at room temperature for 30 min, each at a final concentration of 7 µM. Aliquots of 3.5 µl of the incubated sample and TfR1 alone were applied to freshly glow discharged Quantifoil R 1.2/1.3 300 mesh copper grids. The grids were plunged into liquid ethane maintained at liquid nitrogen temperature, using the FEI Vitrobot IV (Thermo Fisher Scientific). The vitrification was performed at room temperature at 100% humidity. The data were collected on Titan Krios G2 (The Hormel Institute) operating at 300 kV equipped with Gatan K4 camera, using nominal magnification of x130,000 at a physical pixel size of 0.66 Å and total dose of 50 e^-^/Å. The data collection resulted in 15,970 and 18,149 movies for the complex and TfR1 alone, respectively.

### Image processing

The image processing for the cryo-EM reconstruction was performed in cryoSPARC v4^41^. Particles were initially picked using a blob picker in cryoSPARC, and selected 2D classes were subsequently used for template-based particle picking. Iterative rounds of 2D classification were performed to remove junk particles from further processing, and an ab-initio model was generated using *C*_2_ symmetry from the selected particle subset. After heterogeneous refinement, 3D density was reconstructed using non-uniform refinement with global and local CTF refinement. Based on resolution improvement, local motion correction was performed for the VP1u-TfR1 complex data, whereas reference-based motion correction was performed for the TfR1 alone data. The motion-corrected particles were re-refined, yielding 2.23 Å and 2.42 Å resolution for VP1u-TfR1 and TfR1-alone, respectively.

To resolve partial occupancy of VP1u on the dimeric TfR1, symmetry expansion followed by focused 3D classification was performed. The 681,957 particles refined with *C*_2_ symmetry were symmetry-expanded yielding 1,363,914 particles. Using a mask focused on one of the VP1u densities, 3D classification without alignment was performed to discard particles lacking VP1u. The same process was repeated for the other VP1u density on TfR1, yielding 1,017,894 particles. To account for the flexible interaction between VP1u and TfR1, the particles were subjected to flexible refinement, including 3D flex data prep, mesh prep, training, and reconstruction, yielding a final map at 2.41 Å resolution.

### Model building and refinement

The atomic model of the human TfR1^23^ was initially refined against the cryo-EM density of the TfR1-alone map by using ISOLDE^42^ and real-space refinement in PHENIX^43^. For the atomic model of VP1u, the density corresponding to VP1u was isolated from the VP1u-TfR1 complex map by applying a mask and, together with amino acid sequences of VP1u or VP1, was subjected to ModelAngelo^24^ in Relion 5.0 for de novo model building. The generated model for the VP1u RBD was refined independently by using ISOLDE^42^, combined with TfR1, and further refined against the VP1u-TfR1 complex map using ISOLDE^42^ and real-space refinement in PHENIX^43^. Final validation for the models was done using MolProbity^44^.

### Structural analysis and display

Contacts between VP1u RBD and TfR1 were identified as atom pairs within the van der Waals contact distance (sum of atomic radii plus a 0.4 Å tolerance) in ChimeraX^45^. Map visualization and images were also generated in ChimeraX^45^.

### Quantification and statistical analysis

Statistical analyses were performed using GraphPad Prism version 10.3.1 (GraphPad Software, Boston, MA, USA). The specific statistical tests applied are indicated in the corresponding figure legends. Error bars represent standard deviation (SD), as specified in each legend. A *p* value < 0.05 was considered statistically significant in all analyses.

### Reporting summary

Further information on research design is available in the Nature Portfolio Reporting Summary linked to this article.

## Supplementary information

Supplementary information

Reporting summary

Transparent Peer Review file

## Source data

Source Data

## Data Availability

The mass spectrometry proteomics data generated in this study have been deposited to the ProteomeXchange Consortium via the PRIDE^46^ partner repository with the dataset identifier PXD076389. (http://proteomecentral.proteomexchange.org/cgi/GetDataset?ID=PXD076389). The cryo-EM maps and atomic coordinates of the TfR1 alone and the VP1u-TfR1 complex have been deposited in the EM database (https://www.emdatabank.org) and protein data bank (https://www.rcsb.org) under accession codes EMD-75944 (PDB entry 11QC)[10.2210/pdb11QC/pdb] and EMD-75980 (PDB entry 11RN)[10.2210/pdb11RN/pdb], respectively. Source data are provided with this paper.
