# Supplementary material for: Transferrin receptor 1 binds human parvovirus B19 VP1u to facilitate entry
Source: Nat Commun. 2026 Jun 11;17:7443. doi: 10.1038/s41467-026-74283-7 (PMC13408166; doi:10.1038/s41467-026-74283-7)
Supplement: Supplementary file 1 — Supplementary information [file 41467_2026_74283_MOESM1_ESM.pdf]

Supplementary information

Table of Contents

Supplementary Figure 1. Schematic representation of the Selective Proteomic Proximity Labeling Assay Using Tyramide (SPPLAT).

Supplementary Figure 2. Mass photometry of the VP1u-TfR1 complexes.

Supplementary Figure 3. Binding kinetics of TfR1 with recombinant VP1u monomer and dimer.

Supplementary Figure 4. Binding and uptake of recombinant VP1u monomer and dimer in UT7/Epo cells.

Supplementary Figure 5. Schematic representation of the cryo-EM single particle analysis of the VP1u-TfR1 complex.

Supplementary Figure 6. Single particle analysis of the VP1u-TfR1 complex and TfR1 alone.

Supplementary Figure 7. Schematic representation of the cryo-EM single particle analysis of TfR1.

Supplementary Figure 8. Local resolution maps.

Supplementary Figure 9. Comparisons of the TfR1 structures.

Supplementary Figure 10. AlphaFold3 model of the VP1u.

Supplementary Table 1. Cryo-EM data collection, processing, refinement and validation statistics.

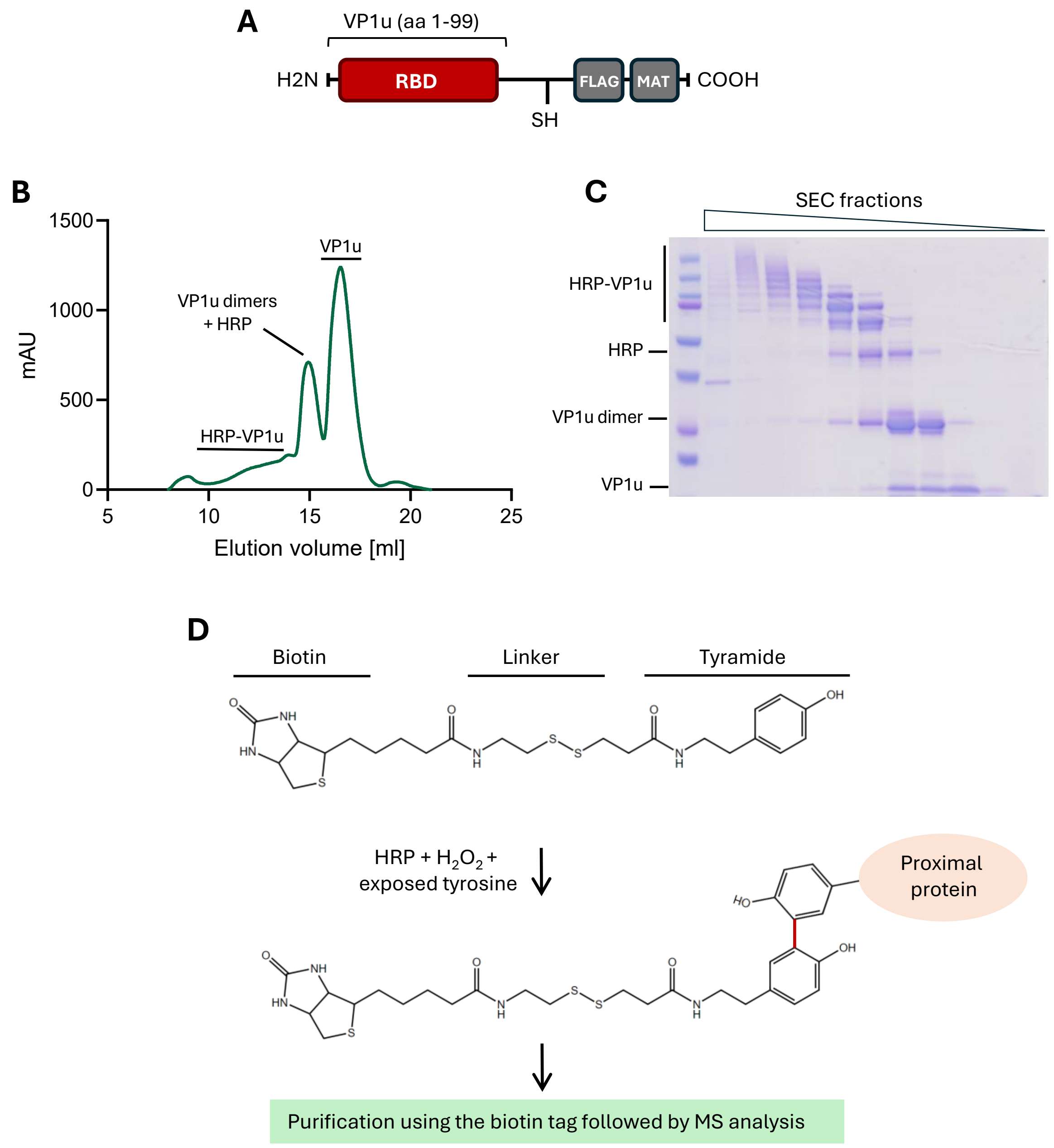

Supplementary Figure 1. Schematic representation of the Selective Proteomic Proximity Labeling Assay Using Tyramide (SPPLAT). (A) Schematic depiction of the recombinant VP1u with a 128 amino acid truncation at its C-terminus. After conjugation of VP1u molecules to HRP, the reactants were separated using size-exclusion chromatography (SEC) (B) and collected fractions were analyzed by SDS-PAGE (C). (D) Schematic illustration of the SPPLAT. HRP generates reactive tyramide radicals in the presence of H_2O_2, leading to covalent biotinylation of proteins in close proximity at the plasma membrane. Source data are provided as a Source Data file.

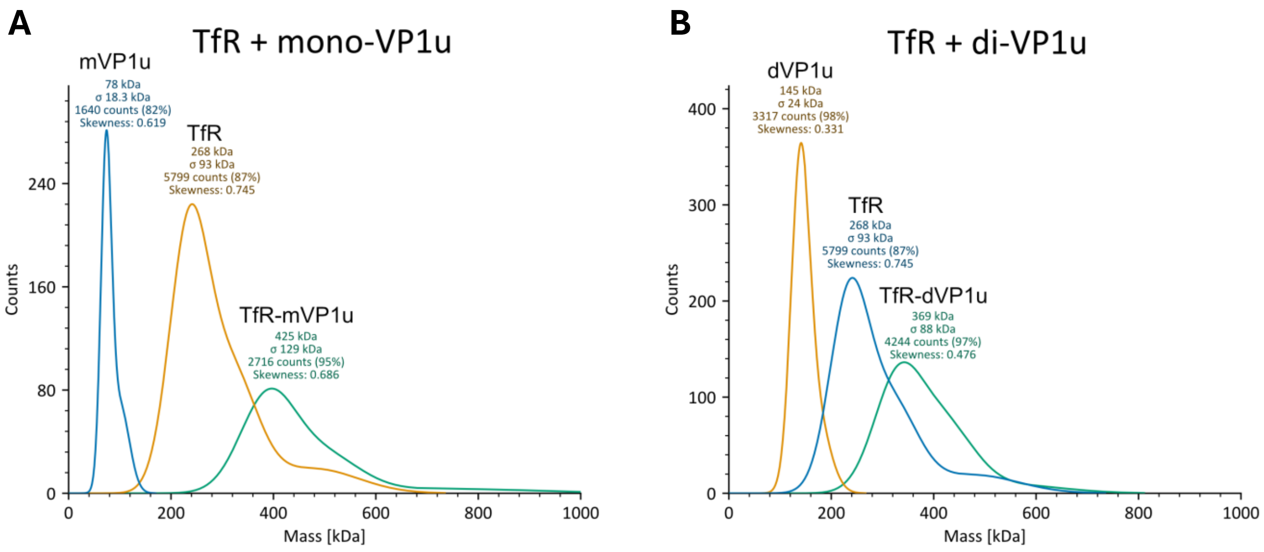

Supplementary Figure 2. Mass photometry of the VP1u-TfR1 complexes. The mass photometry histograms of TfR1, VP1u recombinants, and their mixtures show the molecular size distributions and the formation of TfR1-VP1u complexes for the VP1u monomer **(A)** and VP1u dimer **(B)**.

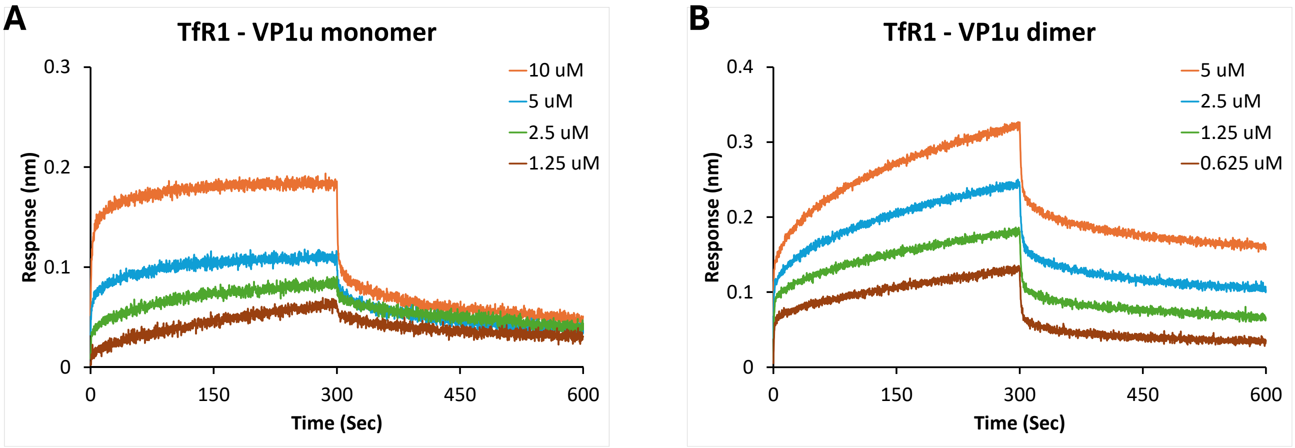

C

Analyte	VP1u monomer	VP1u dimer
K_D1 (M)	$3.06 \pm 0.04 \times 10^{-7}$	$2.61 \pm 0.11 \times 10^{-7}$
K_D2 (M)	$3.69 \pm 0.36 \times 10^{-7}$	$4.48 \pm 0.06 \times 10^{-7}$
Ka1 (1/Ms)	$5.15 \pm 0.05 \times 10^{3}$	$8.27 \pm 0.33 \times 10^{5}$
Ka2 (1/Ms)	$7.28 \pm 0.69 \times 10^{5}$	$1.75 \pm 0.02 \times 10^{3}$
Kd1 (1/s)	$1.57 \pm 0.01 \times 10^{-3}$	$2.16 \pm 0.03 \times 10^{-1}$
Kd2 (1/s)	$2.69 \pm 0.05 \times 10^{-1}$	$7.86 \pm 0.07 \times 10^{-4}$
RSS	0.2424	0.4063
R^2	0.9897	0.9932

*RSS: residual sum of squares

Supplementary Figure 3. Binding kinetics of TfR1 with recombinant VP1u monomer and dimer. Binding of VP1u monomer **(A)** and dimer **(B)** to TfR1-Fc was measured by bio-layer interferometry using serial dilutions. TfR1-Fc was immobilized on an anti-Fc biosensor surface. VP1u samples were incubated for 300 s, followed by 300 s dissociation phase. **(C)** Binding kinetics parameters are summarized in the table. Two binding modes, K_D1 and K_D2, were analyzed using a 2:1 heterogeneous ligand model.

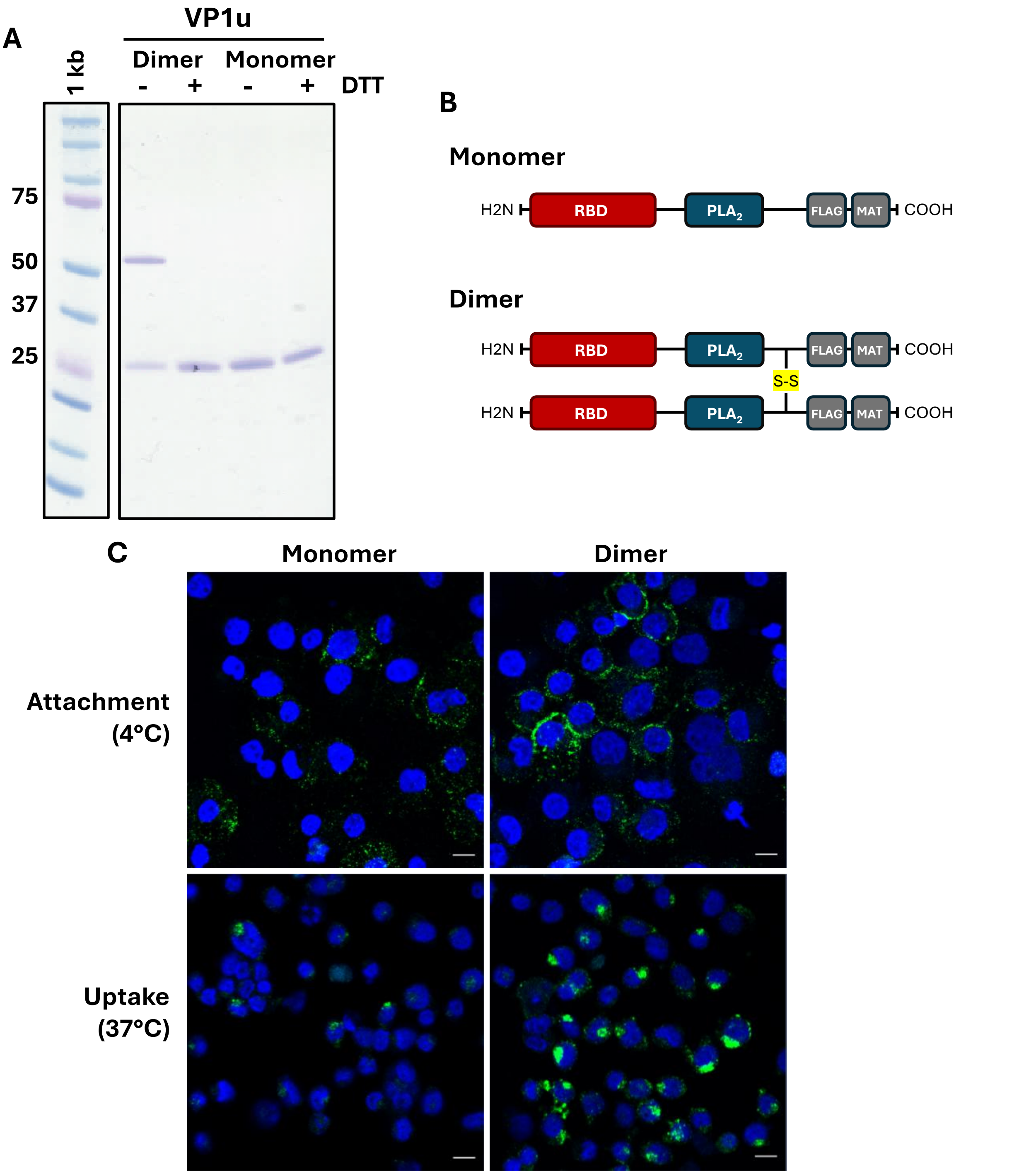

Supplementary Figure 4. Binding and uptake of recombinant VP1u monomer and dimer in UT7/Epo cells. (A) SDS-PAGE analysis of purified recombinant VP1u proteins (monomer and dimer) used in this study. Molecular weight markers are shown on the left. Purified VP1u preparations were separated by electrophoresis and visualized by Coomassie staining. **(B)** Schematic representation of the VP1u monomer and dimer. The N-terminal receptor-binding domain (RBD) and the phospholipase A_2 (PLA_2) domain are indicated. Constructs contain C-terminal FLAG and MAT tags. One construct contains an engineered disulfide bond (S-S) to generate a covalent VP1u dimer. **(C)** Confocal microscopy images of UT7/Epo cells incubated with recombinant VP1u constructs. Cells were exposed to monomer or dimer VP1u proteins under conditions allowing cell surface binding (4 °C) and uptake (37 °C) and subsequently analyzed by confocal microscopy. Scale bar: 10 µm. The experiment was independently repeated twice with similar results. Source data are provided as a Source Data file.

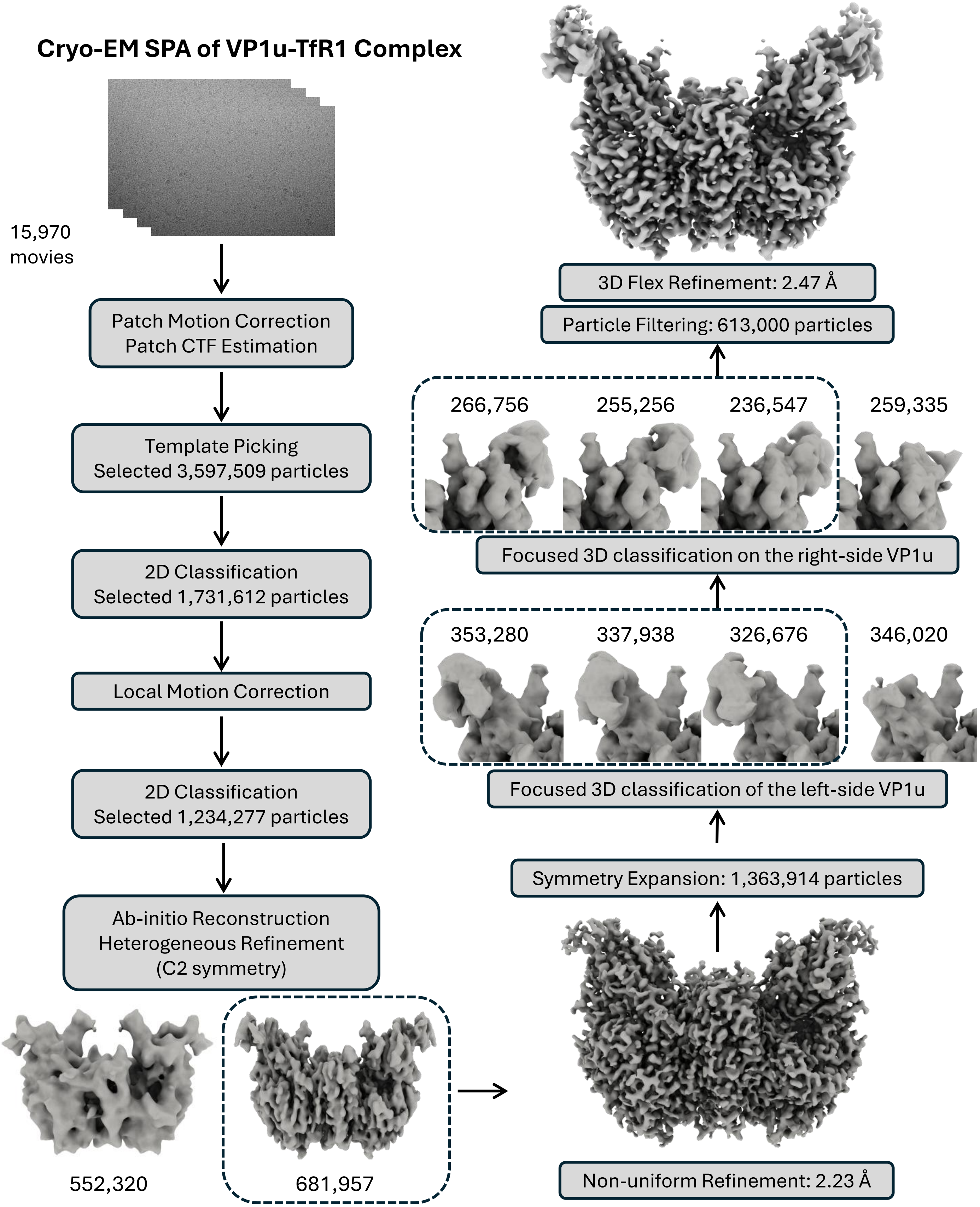

Supplementary Figure 5. Schematic representation of the cryo-EM single particle analysis of the VP1u-TfR1 complex.

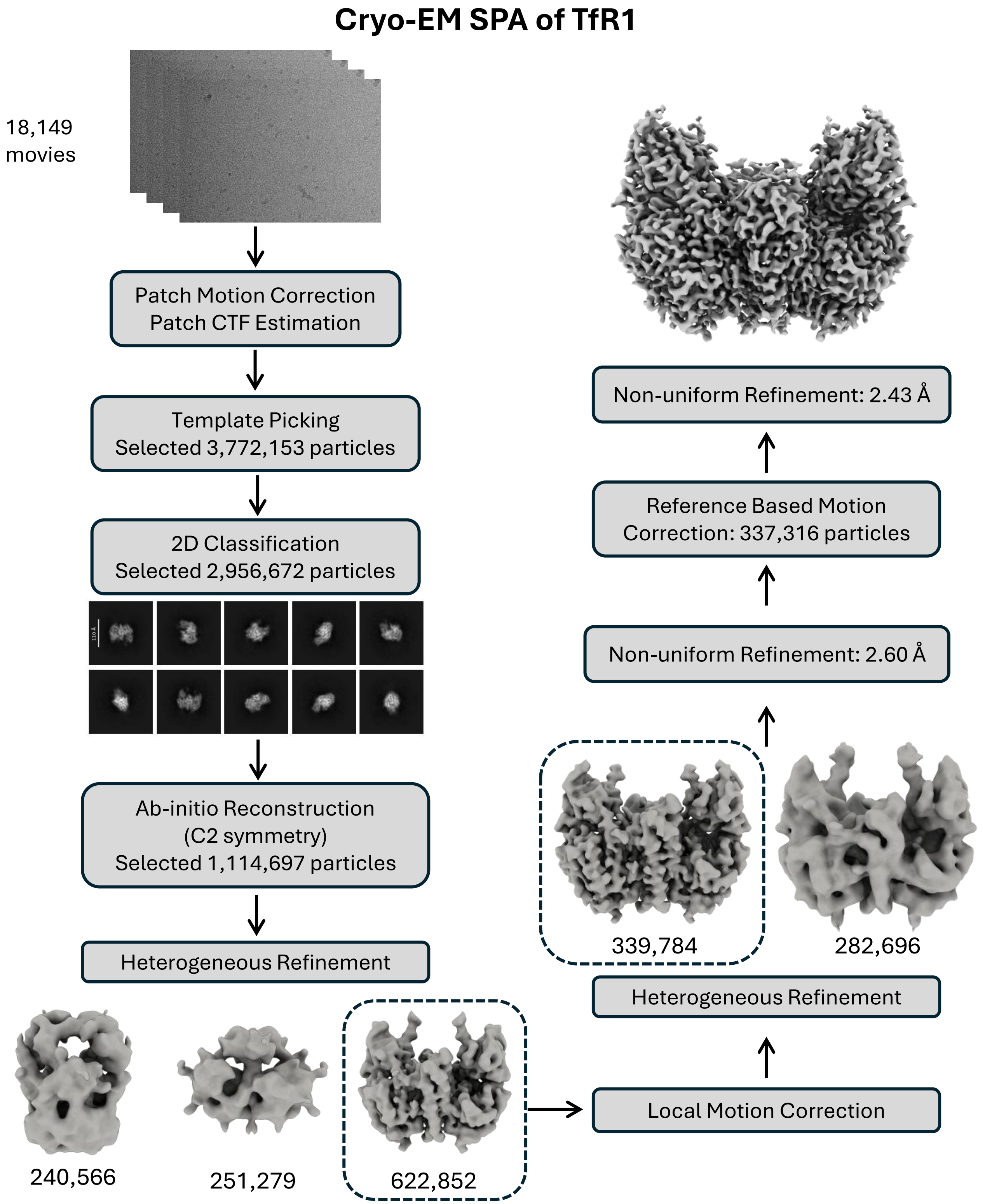

Supplementary Figure 6. Single particle analysis of the VP1u-TfR1 complex and TfR1 alone. Global resolution estimates based on the gold standard Fourier Shell Correlation (GSFSC)(0.143 cutoff) for the 3D reconstructions of the VP1u-TfR1 complex **(A)**, TfR1 alone **(B)**, and the 3D flex reconstruction of the VP1u-TfR1 complex **(C)**. Corresponding particle viewing-direction distribution plots are shown in the right panels. **(D)** Quality of the protein model built into the representative cryo-EM map density (transparent grey)(A to C, left to right). Residues 338-349 within the TfR1 apical domain are shown for each structure.

VP1u-TfR1 Complex

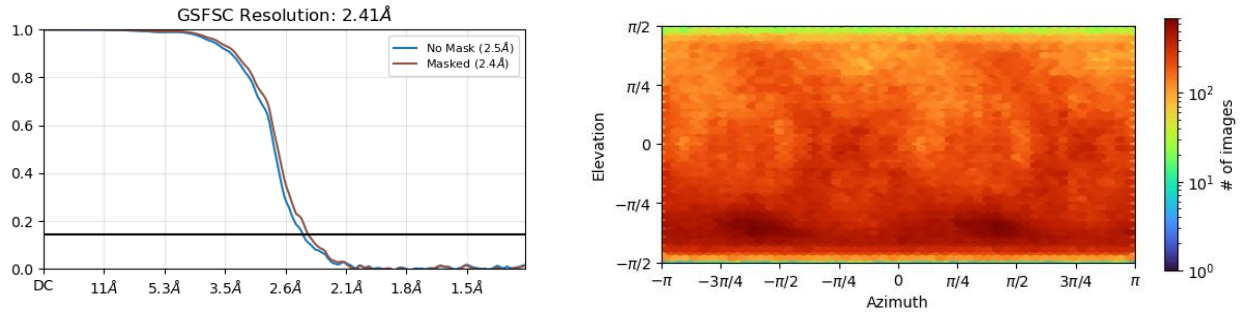

TfR1

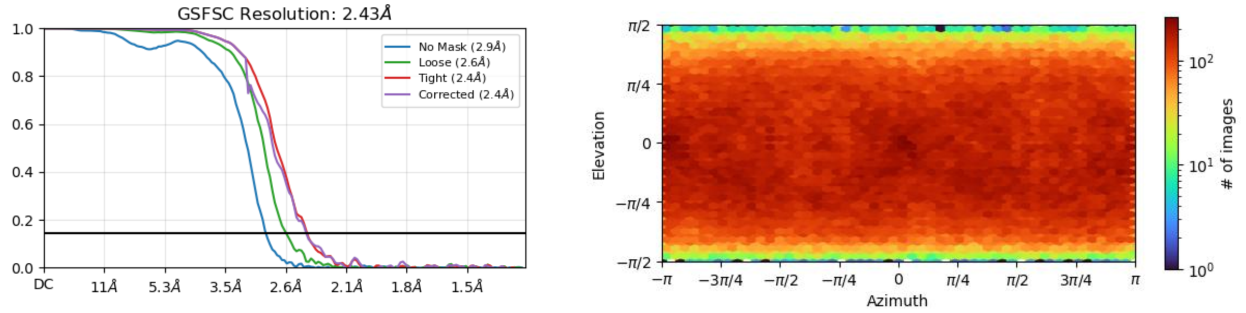

Supplementary Figure 7. Schematic representation of the cryo-EM single particle analysis of TfR1.

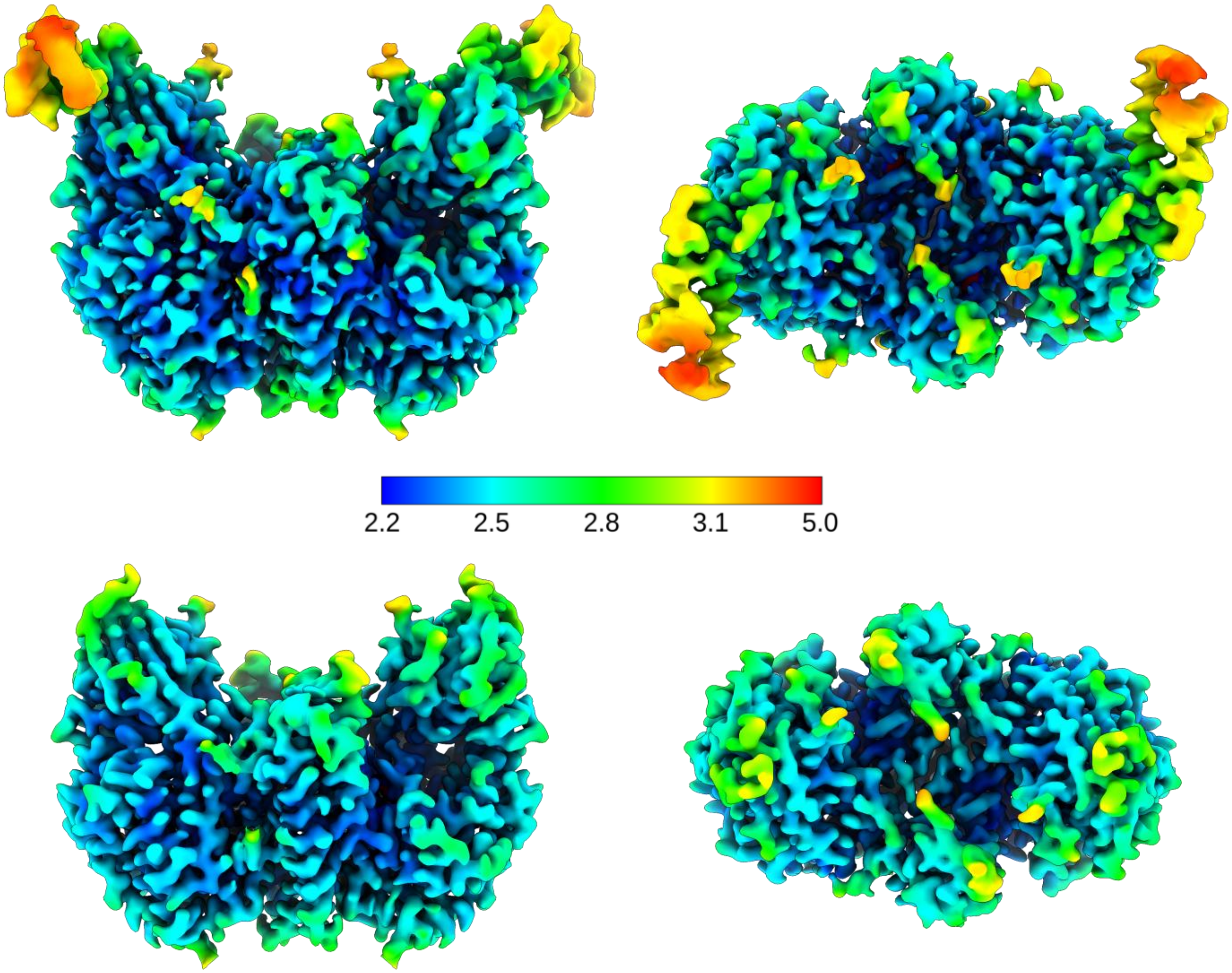

Supplementary Figure 8. Local resolution maps. The local resolutions of the VP1u-TfR1 and TfR1-alone cryo-EM maps are shown. The rendered surfaces are color-coded according to the key in the panel**.**

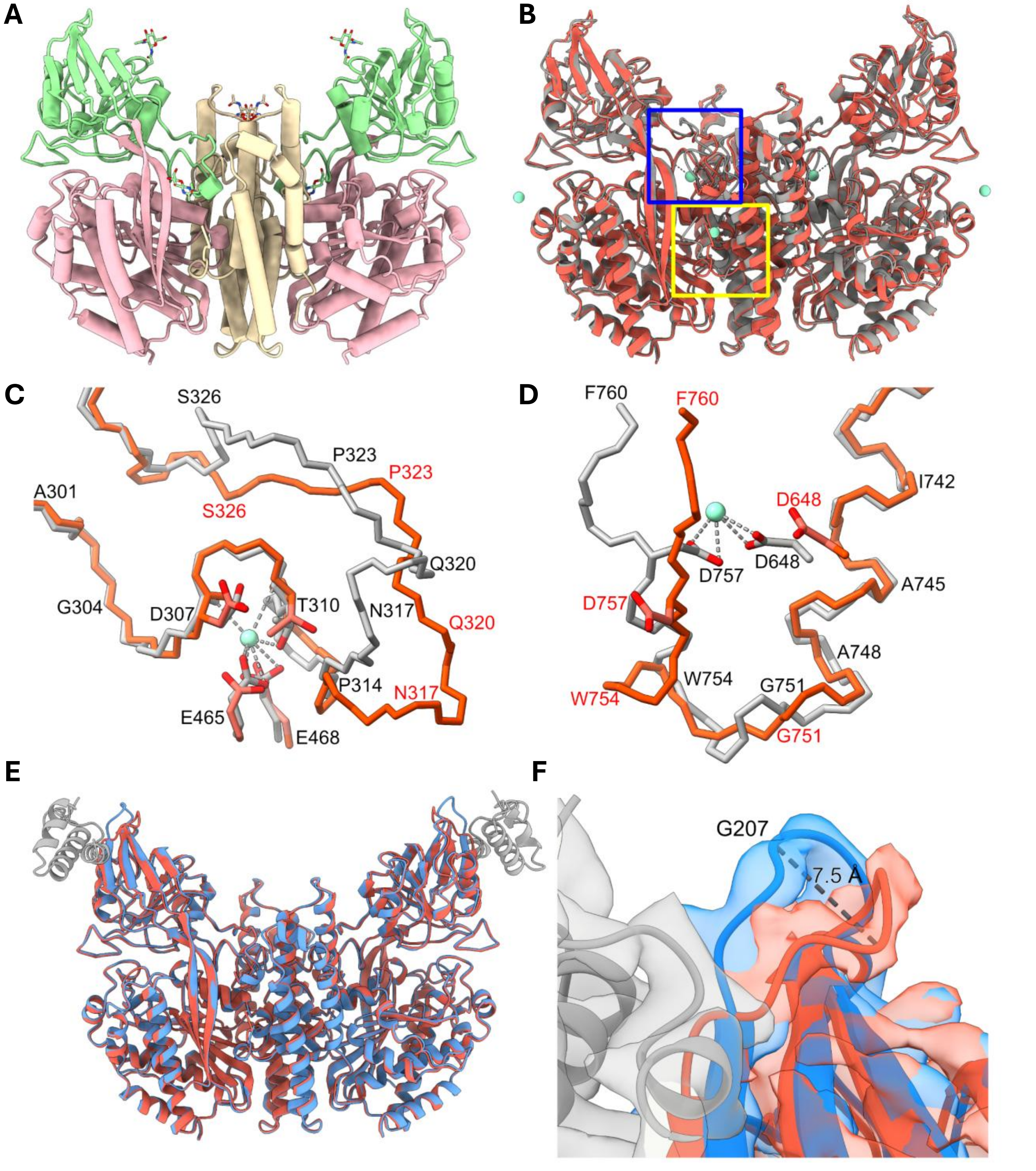

Supplementary Figure 9. Comparisons of the TfR1 structures. (A) Atomic model of human TfR1 generated from the cryo-EM structure. Domains are colored as in Fig. 5. **(B-D)** The cryo-EM structure (red) aligned with human TfR1 crystal structure (gray)(pdb_00001cx8)[https://doi.org/10.2210/pdb1CX8/pdb]. Sm^{3+} ions are shown in light green spheres. Regions highlighted by blue and yellow boxes are zoomed in panels C and D, respectively. **(C)** Conformational differences in the apical traverse loop (residues Pro 314–Gly 328). **(D)** Conformational variation in the C-terminal residues. **(E)** Comparison of the TfR1-alone structure (red) with the VP1u-TfR1 complex (blue). **(F)** Close-up view of the apical loop, showing the conformational change upon RBD binding (gray).

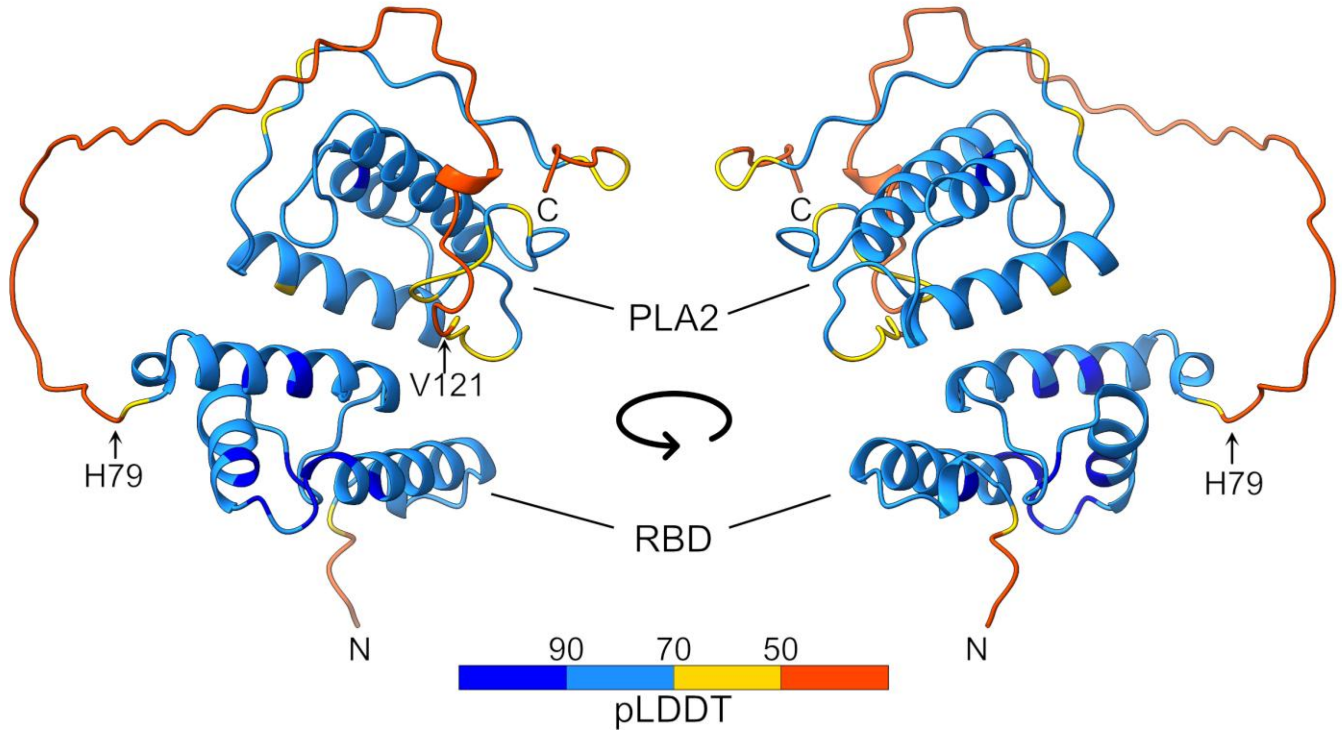

Supplementary Figure 10. AlphaFold3 model of the VP1u. The Alphafold3 prediction of the VP1u (residues 1-227) is shown as a ribbon diagram, colored by predicted local distance difference test (pLDDT) according to the key. The RBD and PLA domains are labeled.

	TfR1	VP1u-TfR1
EMDB	EMD-75944	EMD-75980
PDB	11QC	11RN
**Data collection and processing**		
Magnification	130Kx	130Kx
Voltage (kV)	300	300
Electron exposure (e–/Å^2)	50	50
Defocus range (μm)	-1.0 to -2.0	-1.0 to -2.0
Pixel size (Å)	0.66	0.66
Initial micrographs (no.)	18,145	15,696
Final micrographs (no.)	17,310	15,639
Symmetry imposed	C2	C2 (SymExp)
Particles (no.)	337,316	613,000
Map resolution (Å)	2.43	2.41
**Refinement**		
Initial model used (PDB code)	1CX8	11QC
Model resolution (Å)	2.4	2.2
FSC threshold	0.143	0.143
Model resolution range (Å)	2.4 – 2.5	2.1 – 2.5
Map sharpening B factor (Å^2)	103.0	NA
Model composition		
Non-hydrogen atoms	10,198	11,202
Protein residues	1,278	1,398
B factors (Å^2)		
Protein	36.76	72.51
R.m.s. deviations		
Bond lengths (Å)	0.003	0.003
Bond angles (°)	0.694	0.657
Validation		
MolProbity score	0.94	1.02
Clashscore	0.79	1.04
Poor rotamers (%)	0.55	1.00
Ramachandran plot		
Favored (%)	96.86	96.62
Allowed (%)	2.98	3.24
Disallowed (%)	0.16	0.14

Supplementary Table 1. Cryo-EM data collection, processing, refinement and validation statistics.
