# Supplementary material for: Transferrin receptor 1 binds human parvovirus B19 VP1u to facilitate entry
Source: Nat Commun. 2026 Jun 11;17:7443. doi: 10.1038/s41467-026-74283-7 (PMC13408166; doi:10.1038/s41467-026-74283-7)
Supplement: Supplementary file 2 — Reporting summary [file 41467_2026_74283_MOESM2_ESM.pdf]

nature portfolio

Corresponding author(s): Susan Hafenstein
Carlos Roos

Last updated by author(s): May 18, 2026

Reporting Summary

Nature Portfolio wishes to improve the reproducibility of the work that we publish. This form provides structure for consistency and transparency in reporting. For further information on Nature Portfolio policies, see our Editorial Policies and the Editorial Policy Checklist.

Statistics

For all statistical analyses, confirm that the following items are present in the figure legend, table legend, main text, or Methods section.

n/a	Confirmed	
☐	☒	The exact sample size (*n*) for each experimental group/condition, given as a discrete number and unit of measurement
☐	☒	A statement on whether measurements were taken from distinct samples or whether the same sample was measured repeatedly
☐	☒	The statistical test(s) used AND whether they are one- or two-sided<br>*Only common tests should be described solely by name; describe more complex techniques in the Methods section.*
☒	☐	A description of all covariates tested
☐	☒	A description of any assumptions or corrections, such as tests of normality and adjustment for multiple comparisons
☐	☒	A full description of the statistical parameters including central tendency (e.g. means) or other basic estimates (e.g. regression coefficient) AND variation (e.g. standard deviation) or associated estimates of uncertainty (e.g. confidence intervals)
☐	☒	For null hypothesis testing, the test statistic (e.g. *F*, *t*, *r*) with confidence intervals, effect sizes, degrees of freedom and *P* value noted<br>*Give P values as exact values whenever suitable.*
☒	☐	For Bayesian analysis, information on the choice of priors and Markov chain Monte Carlo settings
☒	☐	For hierarchical and complex designs, identification of the appropriate level for tests and full reporting of outcomes
☒	☐	Estimates of effect sizes (e.g. Cohen's *d*, Pearson's *r*), indicating how they were calculated

Our web collection on statistics for biologists contains articles on many of the points above.

Software and code

Policy information about availability of computer code

Data collection	Confocal microscopy images were acquired using ZEISS ZEN software on a ZEISS LSM880 laser scanning confocal microscope.<br>MaxQuant v2.0.1.0 was used for mass spectrometry data analysis.<br>EPU (E pluribus unum) commercial software on Krios microscope used to collect data.
Data analysis	Statistical analyses and graphical representations were performed using GraphPad Prism (version 10.3.1). Quantitative PCR data were analyzed using the instrument software and GraphPad Prism. Mass spectrometry data were processed and quantified by the proteomics facility using standard peptide intensity-based quantification workflows.<br>CryoSparc is freeware used to reconstruct the cryo EM data.

For manuscripts utilizing custom algorithms or software that are central to the research but not yet described in published literature, software must be made available to editors and reviewers. We strongly encourage code deposition in a community repository (e.g. GitHub). See the Nature Portfolio guidelines for submitting code & software for further information.

Data

Policy information about availability of data

All manuscripts must include a data availability statement. This statement should provide the following information, where applicable:

- Accession codes, unique identifiers, or web links for publicly available datasets
- A description of any restrictions on data availability
- For clinical datasets or third party data, please ensure that the statement adheres to our policy

The mass spectrometry proteomics data generated in this study have been deposited to the ProteomeXchange Consortium via the PRIDE47 partner repository with the dataset identifier PXD076389. (http://proteomecentral.proteomexchange.org/cgi/GetDataset?ID=PXD076389).The cryo-EM maps and atomic coordinates of the TfR1 alone and the VP1u-TfR1 complex have been deposited in the EM database (https://www.emdatabank.org) and protein data bank (https://www.rcsb.org) under accession codes EMD-75944 (PDB entry 11QC) and EMD-75980 (PDB entry 11RN), respectively. Source data are provided with this paper.

Research involving human participants, their data, or biological material

Policy information about studies with human participants or human data. See also policy information about sex, gender (identity/presentation), and sexual orientation and race, ethnicity and racism.

Reporting on sex and gender	n.a.
Reporting on race, ethnicity, or other socially relevant groupings	n.a.
Population characteristics	n.a.
Recruitment	n.a.
Ethics oversight	n.a.

Note that full information on the approval of the study protocol must also be provided in the manuscript.

Field-specific reporting

Please select the one below that is the best fit for your research. If you are not sure, read the appropriate sections before making your selection.

☒ Life sciences ☐ Behavioural & social sciences ☐ Ecological, evolutionary & environmental sciences

For a reference copy of the document with all sections, see nature.com/documents/nr-reporting-summary-flat.pdf

Life sciences study design

All studies must disclose on these points even when the disclosure is negative.

Sample size	Sample sizes were determined based on established experimental procedures for virus binding, uptake, and infection assays in cultured cells. Experiments were performed using independent biological replicates (n ≥ 3 per condition).
Data exclusions	No data were excluded unless technical failure occurred (for example failed PCR amplification or imaging artifacts preventing interpretation).
Replication	All experiments were independently repeated at least three times with consistent results.
Randomization	n.a.
Blinding	n.a.

Reporting for specific materials, systems and methods

We require information from authors about some types of materials, experimental systems and methods used in many studies. Here, indicate whether each material, system or method listed is relevant to your study. If you are not sure if a list item applies to your research, read the appropriate section before selecting a response.

Materials & experimental systems

n/a	Involved in the study	
☐	☒	Antibodies
☐	☒	Eukaryotic cell lines
☒	☐	Palaeontology and archaeology
☒	☐	Animals and other organisms
☒	☐	Clinical data
☒	☐	Dual use research of concern
☒	☐	Plants

Methods

n/a	Involved in the study	
☒	☐	ChIP-seq
☒	☐	Flow cytometry
☒	☐	MRI-based neuroimaging

Antibodies

Antibodies used	Anti-human transferrin receptor 1 monoclonal antibody OKT9 (Caprico Biotechnologies); rabbit anti-FLAG antibody (Cell Signaling Technology); mouse anti-human TfR1 antibody recognizing residues 3–28 of the cytoplasmic tail (Thermo Fisher Scientific); monoclonal antibody against parvovirus B19 capsid protein VP2 (Mikrogen); Alexa Fluor–conjugated secondary antibodies (Thermo Fisher Scientific / Invitrogen).
Validation	All antibodies are commercially available and validated by manufacturers for immunofluorescence or blocking assays. Functional validation of the TfR1 blocking antibody OKT9 was confirmed experimentally by demonstrating inhibition of VP1u binding and virus internalization in cell-based assays.

Eukaryotic cell lines

Policy information about cell lines and Sex and Gender in Research

Cell line source(s)	UT7/Epo cells (provided by E. Morita, Tohoku University School of Medicine).<br>KU812Ep6 cells derived from KU812 erythroleukemia cells (ATCC; CRL-2099)<br>Jurkat cells (ATCC; TIB-152).<br>HepG2 cells (ATCC; HB-8065).<br>HeLa cells (ATCC; CCL-2).
Authentication	Cell lines were obtained from established sources and maintained according to standard cell culture procedures. Identity was verified based on supplier documentation and expected morphology and growth characteristics.
Mycoplasma contamination	Cells were maintained under sterile culture conditions and periodically tested to confirm absence of mycoplasma contamination.
Commonly misidentified lines<br>(See ICLAC register)	All cell lines used are widely established and were handled following standard laboratory procedures to prevent cross-contamination.

Plants

Seed stocks	n.a.
Novel plant genotypes	n.a.
Authentication	n.a.
