# Supplementary material for: Transferrin receptor 1 binds human parvovirus B19 VP1u to facilitate entry
Source: Nat Commun. 2026 Jun 11;17:7443. doi: 10.1038/s41467-026-74283-7 (PMC13408166; doi:10.1038/s41467-026-74283-7)
Supplement: Supplementary file 3 — Transparent Peer Review file [file 41467_2026_74283_MOESM3_ESM.pdf]

nature portfolio

Peer Review File

Transferrin receptor 1 binds human parvovirus B19 VP1u to facilitate entry

Corresponding Author: Professor Carlos Ros

This file contains all reviewer reports in order by version, followed by all author rebuttals in order by version.

Version 0:

Reviewer comments:

Reviewer #1

(Remarks to the Author)
The manuscript by Lee, Bieri et al. details the identification and characterization of the previously unknown receptor of human parvovirus B19, which interacts with the externalized VP1u region of the virus. The authors show that this interaction is essential for the internalization of B19V, but not for virus binding. The virus-receptor interaction is of protein-protein in nature and occurs without the involvement of cell surface glycans. Despite of TfR1 being a common cell surface protein, viral entry only takes place in erythroid progenitor-like cell lines. Structural reconstruction of the VP1u-TfR1 interaction verifies the importance of residues, which were previously deemed essential for B19V entry. Overall, the manuscript presents a well-written and well-executed study, which is of great interest to virologists regardless of field of study. Only few questions and comments remain to be addressed by the authors, as follows:

It is suggested that VP1u dimerization facilitates a more efficient engagement with the TfR1 dimer. VP1 is a minor capsid protein and is incorporated to the viral capsid in a 1:20 ratio, which would mean only three VP1 per capsid. Although B19V exhibits a rather large VP1u, it is still difficult to imagine how dimerization would happen, even in case of neighboring fivefold axes. Consequently, the biological relevance of this finding may be questioned and requires further investigations. Could the two VP1u domains within the dimer be donated by two separate virions?

The residues belonging to the PLA2 domain and other subsequent VP1u regions were not ordered in the C2 reconstructions. Was it verified that these domains of a highly helical disposition were correctly folded after the recombinant protein expression?

Line 387: what type of cellular context is referred to here that would explain the erythroid progenitor-specificity?

Figure 1A: Without labeling the bands in the SDS-PAGE it is difficult to interpret how each band contributes to the verification of the maleimide-conjugation and expression of HRP-VP1u.

Figure 4: The density map appears to have lower resolution than the authors claim, based on the density associated with the sidechains, especially in the VP1u RBD. It would be informative to include a local resolution map so that the sidechain orientations could be interpreted in the context of the actual local resolution.

Reviewer #2

(Remarks to the Author)
This is an advanced finding on TfR1 (CD71) in VP1u-mediated B19V entry in erythroid cell lines. And, TfR1 is the receptor engaged to VP1u to trigger B19V internalization.
However, some conclusions require resolution through TfR1 knockdown in the erythroid cell line.

Further, in the experiment of Fig 4. VP1u binding was detected exclusively in KU812Ep6 cells and remained undetectable in non-erythroid cells under identical conditions. The findings indicate that the lack of VP1u binding in non-erythroid cells

cannot be attributed to the absence of TfR1 at the cell surface and instead point to an erythroid-specific context required for productive VP1u-TfR1 interaction. Please explain and discuss in detail.

And as we know, TfR1 is also an important entry factor for JEV, SARS-CoV-2, and other viruses. Based on your results, please discuss this.

Reviewer #3

(Remarks to the Author)
Determining the molecular identity of a human pathogen is typically a highly significant step in the evolution of our understanding of that pathogen, and structural characterization of the pathogen/host interaction is an important followup for countermeasures development and/or for fundamental understanding of the host-pathogen interaction. B19 parvovirus is a huge pain to work with and therefore our understanding has improved at a glacial pace compared to other human pathogens. This work is therefore likely to be significant in the field, and is presaged by other works of Ros and peer investigators that built up to this discovery. The study is well fleshed-out with multiple orthogonal approaches.

I have no substantive scientific criticisms of the manuscript. The experiments are sound, particularly the structural experiments which I am most qualified to assess.

Minor comments:
Section "TfR1 glycosylation does not affect VP1u uptake in erythroid cells": TfR is also O-glycosylated with biologically-significant mucin glycosylation. O-glycosidase treatment is mentioned in the Methods section but not in the Results section here. Is this because of the known issues with O-glycosidase not removing all O-linked glycans? I seem to recall that there is circumstantial evidence that mucin-like glycosylation, found in the stalk domain, may be relevant to entry of certain viruses, but that literature is distant to me.

L. 302: Is the occupancy approximately equal in the VP1u region and the apical domain region?

An essential literature omission is "Dual Host-Virus Arms Races Shape an Essential Housekeeping Protein" by Demogines et al.

l. 395: Should also cite the AAV variants binding TfR

l. 404 "the" entry receptor is an overclaim from these data.

l. 570: Are both moieties at 7μM? If not, what is the concentration of each?

l. 581-582: Blob picker outputs aren't an input to template picking. Do you mean to say that selected 2D classes of the blob picks were used for template picking?

l. 591 In Schoenflies notation, the C should be italicized and the 2 subscripted.

l. 597 The meaning of FSC=0.143 from 3D Flex is unclear because of algorithmic biases in neural network based methods operating on the density. It would be useful to also report the best resolution that could be obtained without neural network and the estimated local resolution by eye.

l. 609 "separated by less than 0.4-Å van der Waal's radius" I think you are meaning that compute the distance between the atom centers and subtract the vdW radii of each atom and consider them interacting if the residual is less than 0.4. Is this correct? The line should be rephrased for clarity.

l. 615 italicize p

Fig 1D This presentation is less informative. Perhaps we could get scores in the table? I assume these are the only ones on the list because of a cutoff used: was it a Z-score, a certain log2FC threshold, etc?

Fig 2 Preferred to use colorblind-safe colors

Fig 6 B-C It would be useful to have a transparent isosurface overlay so we know the extent to which these side-chain-level interactions are supported by density vs. inferred by overall chain placement

Extended Fig 7 C-D At the reported resolution, it would be more scientifically meaningful to use an atom depiction instead of a ribbon depiction.
7F Why are both transparent densities the same base color?

I would recommend adding the 2.23Å NU-refinement map as an "Additional map" to the EMDB deposition because of the ongoing uncertainties around how to interpret 3DFlex densities.

Take a look at the Asn/Gln/His flips on the deposited models.

Version 1:

Reviewer comments:

Reviewer #1

(Remarks to the Author)
The authors sufficiently addressed all my concerns and comments. The new edits have significantly improved the manuscript by making the findings easier to interpret and by providing additional context when discussing the results. The additional supplementary figures have significantly improved the presentation of the results. This will be of great interest for the structural-, clinical- and parvovirus fields. I have no further comments.

Reviewer #2

(Remarks to the Author)
In the discussion of the revised manuscript, there is only text and citations. Please discuss in depth the "TfR1 entry factor " shared by those viruses (non-erythroid cells) and B19V (erythroid cells).

Open Access This Peer Review File is licensed under a Creative Commons Attribution 4.0 International License, which permits use, sharing, adaptation, distribution and reproduction in any medium or format, as long as you give appropriate credit to the original author(s) and the source, provide a link to the Creative Commons license, and indicate if changes were made.
In cases where reviewers are anonymous, credit should be given to 'Anonymous Referee' and the source.
The images or other third party material in this Peer Review File are included in the article's Creative Commons license, unless indicated otherwise in a credit line to the material. If material is not included in the article's Creative Commons license and your intended use is not permitted by statutory regulation or exceeds the permitted use, you will need to obtain permission directly from the copyright holder.
To view a copy of this license, visit https://creativecommons.org/licenses/by/4.0/

Nature Communications manuscript NCOMMS-26-021762-T

REVIEWER COMMENTS (black font)

Point by point response (blue)

Reviewer #1 (Remarks to the Author):

The manuscript by Lee, Bieri et al. details the identification and characterization of the previously unknown receptor of human parvovirus B19, which interacts with the externalized VP1u region of the virus. The authors show that this interaction is essential for the internalization of B19V, but not for virus binding. The virus-receptor interaction is of protein-protein in nature and occurs without the involvement of cell surface glycans. Despite of TfR1 being a common cell surface protein, viral entry only takes place in erythroid progenitor-like cell lines. Structural reconstruction of the VP1u-TfR1 interaction verifies the importance of residues, which were previously deemed essential for B19V entry. Overall, the manuscript presents a well-written and well-executed study, which is of great interest to virologists regardless of field of study. Only few questions and comments remain to be addressed by the authors, as follows:

It is suggested that VP1u dimerization facilitates a more efficient engagement with the TfR1 dimer. VP1 is a minor capsid protein and is incorporated to the viral capsid in a 1:20 ratio, which would mean only three VP1 per capsid. Although B19V exhibits a rather large VP1u, it is still difficult to imagine how dimerization would happen, even in case of neighboring fivefold axes. Consequently, the biological relevance of this finding may be questioned and requires further investigations. Could the two VP1u domains within the dimer be donated by two separate virions?

> We understand the Reviewer's concern, but the available structural data indicate that a same-virion scenario is geometrically feasible.
>
> Our cryo-EM structure shows that one TfR1 dimer can bind two VP1u RBDs, one on each apical domain. The distance across these two binding sites is on the order of ~90 Å.
>
> From the known B19V capsid geometry (~260 Å diameter), the distance between adjacent fivefold axes is ~120-130 Å. This closely matches the span of the two apical binding sites on TfR1. Therefore, two VP1u molecules externalized from neighboring fivefolds on the same capsid could, in principle, simultaneously engage the two apical domains of one TfR1 dimer.
>
> A same-fivefold model is also possible: VP1u is 227 aa long, which provides sufficient reach for two VP1u molecules emerging from the same fivefold to span the ~80-130 Å separation on TfR1.
>
> Importantly, our biochemical data do not support strong avidity-driven bivalent engagement (no clear affinity gain for the dimer), indicating that even if such a geometry is possible, it is not required for binding. Thus, while two VP1u domains could originate from the same virion, either from one or two nearby fivefolds, this remains a geometrically plausible but not experimentally demonstrated configuration. Alternatively, the two VP1u molecules bound to one TfR1 dimer could also be contributed by two separate virions.
>
> New lines (412-422) have been added in Discussion.

The residues belonging to the PLA2 domain and other subsequent VP1u regions were not ordered in the C2 reconstructions. Was it verified that these domains of a highly helical disposition were correctly folded after the recombinant protein expression?

To add clarity, we have included Extended Data Figure 9 (also lines 294-299) which shows the alphafold predicted structure of the VP1u and maps the known functional domains. The RBD (residues 5-80) is separated from the PLA (130-195) by about forty-three residues (79-121) which are all unstructured and flexible. Because of the flexible region, there is no possibility of resolving the PLA2, which is too far from the RBD-TfR complex.

The N-terminal RBD is well resolved and adopts the expected three-helix bundle, demonstrating proper folding of the receptor-interacting region. In addition, we have previously mapped the RBD to the N-terminus of VP1u and shown that this region is sufficient for binding and internalization, whereas the PLA2-containing C-terminal portion is dispensable for these processes (Leisi et al., 2013; Leisi et al., 2016; Bircher et al., 2022). Consistent with this, the PLA2 domain lies outside the TfR1-binding interface identified here.

Line 387: what type of cellular context is referred to here that would explain the erythroid progenitor-specificity?

By “cellular context” we refer to how TfR1 is presented at the plasma membrane, including its lateral organization, potential interaction with erythroid-specific co-factors, and local membrane environment. Although TfR1 is broadly expressed, our data indicate that only erythroid cells provide the conditions that permit productive VP1u engagement.

Figure 1A: Without labeling the bands in the SDS-PAGE it is difficult to interpret how each band contributes to the verification of the maleimide-conjugation and expression of HRP-VP1u.

We agree and have revised Fig. 1A to label the bands corresponding to the HRP-VP1u conjugates, and the expected unconjugated HRP.

Figure 4: The density map appears to have lower resolution than the authors claim, based on the density associated with the sidechains, especially in the VP1u RBD. It would be informative to include a local resolution map so that the sidechain orientations could be interpreted in the context of the actual local resolution.

As suggested by the reviewer, we added the local resolution map in the Extended Data Fig. 7. Additionally, Figure 6 has been modified to show isosurface of the map to show the sidechain orientations.

Reviewer #2 (Remarks to the Author):

This is an advanced finding on TfR1 (CD71) in VP1u-mediated B19V entry in erythroid cell lines. And, TfR1 is the receptor engaged to VP1u to trigger B19V internalization. However, some conclusions require resolution through TfR1 knockdown in the erythroid cell line.

We agree that genetic validation would further strengthen the conclusion. However, our identification of TfR1 as the VP1u-engaged entry receptor is supported by multiple independent and complementary approaches, including proximity labeling, colocalization of VP1u with TfR1 at the erythroid cell surface, direct binding in a cell-free system, and functional inhibition experiments. TfR1 is a highly abundant and essential receptor in proliferating erythroid cells, where it plays a central role in iron uptake and cellular metabolism. Its depletion is therefore expected to cause broad effects on cell physiology, including proliferation and viability, which would complicate the interpretation of knockdown experiments in the context of viral entry.

Further, in the experiment of Fig 4. VP1u binding was detected exclusively in KU812Ep6 cells and remained undetectable in non-erythroid cells under identical conditions. The findings indicate that the lack of VP1u binding in non-erythroid cells cannot be attributed to the absence of TfR1 at the cell surface and instead point to an erythroid-specific context required for productive VP1u-TfR1 interaction. Please explain and discuss in detail.

We agree that this point deserves clarification. Our intention was not to imply that the nature of the erythroid-specific context is resolved, but rather to define what our data allow us to exclude and what they suggest. In Fig. 4, non-erythroid cells displayed substantial surface TfR1 but did not bind VP1u, showing that TfR1 expression alone is not sufficient for productive interaction. At the same time, the cryo-EM structure demonstrates that recombinant TfR1 is intrinsically capable of binding VP1u, and glycosidase treatment did not impair VP1u uptake in erythroid cells. Together, these findings argue against models based simply on TfR1 abundance or on an erythroid-specific TfR1 glycoform. Instead, they point to a requirement for the erythroid cellular environment in which TfR1 is presented at the plasma membrane. By “erythroid-specific context,” we refer to factors such as the lateral membrane organization of TfR1, its association with other surface components, or local membrane properties that may determine accessibility or productive engagement by VP1u. Because these determinants were not directly addressed in the present study, we discuss this point cautiously in the Discussion as an unresolved aspect of B19V entry rather than as a defined mechanism.

And as we know, TfR1 is also an important entry factor for JEV, SARS-CoV-2, and other viruses. Based on your results, please discuss this.

We agree that this should be included in our discussion. We have added text and citations in Discussion (lines 399-407).

Reviewer #3 (Remarks to the Author):

Determining the molecular identity of a human pathogen is typically a highly significant step in the evolution of our understanding of that pathogen, and structural characterization of the pathogen/host interaction is an important followup for countermeasures development and/or for fundamental understanding of the host-pathogen interaction. B19 parvovirus is a huge pain to work with and therefore our understanding has improved at a glacial pace compared to other human pathogens. This work is therefore likely to be significant in the field, and is presaged by other works of Ros and peer investigators that built up to this discovery. The study is well fleshed-out with multiple orthogonal approaches.

I have no substantive scientific criticisms of the manuscript. The experiments are sound,

particularly the structural experiments which I am most qualified to assess.

Minor comments:
Section "TfR1 glycosylation does not affect VP1u uptake in erythroid cells": TfR is also O-glycosylated with biologically-significant mucin glycosylation. O-glycosidase treatment is mentioned in the Methods section but not in the Results section here. Is this because of the known issues with O-glycosidase not removing all O-linked glycans? I seem to recall that there is circumstantial evidence that mucin-like glycosylation, found in the stalk domain, may be relevant to entry of certain viruses, but that literature is distant to me.

> We thank the reviewer for this point. O-glycosidase treatment was performed (as described in the Methods) but did not show any detectable effect on VP1u uptake, similar to the other glycosidase treatments, and was therefore not highlighted in the Results. We agree that O-glycosidase has known limitations, particularly for mucin-type O-glycosylation, so incomplete removal cannot be excluded. However, our structural data place the VP1u binding site in the apical domain of TfR1, spatially separated from the glycosylated stalk region, arguing against a direct role of these glycans in the interaction. We added line 204-205 in the Results section for O-glycosidase treatment.

L. 302: Is the occupancy approximately equal in the VP1u region and the apical domain region?

> The text was clarified and more details were added (lines 263-270) to describe that the first NU-refinement map showed lower occupancy of the VP1u region compared to the apical domain. After going through the symmetry expansion and two round of the focused 3D classification and sorting, which removed 55 percents of particles with partial occupancy, the remained particles used in the 3D flex refinement have a full occupancy of the VP1u.

An essential literature omission is "Dual Host-Virus Arms Races Shape an Essential Housekeeping Protein" by Demogines et al.

> We thank the reviewer for pointing out this relevant study. We agree that the work by Demogines et al. highlights the evolutionary importance of TfR1 as a recurrent target of host–virus interactions. We have now incorporated this reference in the Discussion, where we describe the TfR1 apical domain as a common interaction hotspot for diverse pathogens. This provides additional context for our finding that B19V VP1u engages the same region, consistent with the idea that this surface has been shaped by host–virus arms races. Please see Line 403**:** "Consistent with this, the apical domain of TfR1 has been identified as an evolutionary hotspot shaped by host-virus arms races (Demogines et al., 2013)."

l. 395: Should also cite the AAV variants binding TfR

> As suggested by the review, we cited Huang et al.'s recent study of the engineering AAV9 for TfR binding. Please see Line 405**:** "In another parovirus, AAV, engineering the AAV9 capsid to bind the human TfR1 apical domain enabled the vector to actively cross the blood-brain barrier and achieve efficient, brain-wide gene delivery (Huang et al., 2024)."

l. 404 "the" entry receptor is an overclaim from these data.

We agree that the wording “the entry receptor” may be too strong. Our data demonstrate that TfR1 is required for VP1u-mediated uptake and is directly engaged by VP1u to trigger internalization. At the same time, our results also indicate that initial virus attachment occurs independently of TfR1, consistent with a multistep entry mechanism. We have therefore revised the wording throughout the manuscript to more precisely describe TfR1 as a receptor required for VP1u-mediated internalization rather than as the sole entry receptor. Please see Line 430: “…establish TfR1 as the receptor mediating VP1u-dependent internalization into target progenitor cells.”

l. 570: Are both moieties at 7μM? If not, what is the concentration of each?

We thank the reviewer for pointing out the ambiguity in the sentence. It has been revised to specify the concentration of each component.

l. 581-582: Blob picker outputs aren't an input to template picking. Do you mean to say that selected 2D classes of the blob picks were used for template picking?

The sentence has been modified for clarity.

l. 591 In Schoenflies notation, the C should be italicized and the 2 subscripted.

All C2 notation has been modified accordingly (line 255, 610, and 617).

l. 597 The meaning of FSC=0.143 from 3D Flex is unclear because of algorithmic biases in neural network based methods operating on the density. It would be useful to also report the best resolution that could be obtained without neural network and the estimated local resolution by eye.

We agree with the reviewer that the resolution from 3D Flex could be unconventional. Notably, the global resolution of the NU refinement map without neural network was identical to the 3D Flex reconstruction map. As originally mentioned in the manuscript, the VP1u density was weaker and had lower local resolution due to flexibility of the bound VP1u. We added local resolution information in Extended Data Fig. 7.

l. 609 "separated by less than 0.4-Å van der Waal’s radius" I think you are meaning that compute the distance between the atom centers and subtract the vdW radii of each atom and consider them interacting if the residual is less than 0.4. Is this correct? The line should be rephrased for clarity.

We thank the reviewer for pointing out the misleading sentence. We have corrected it to indicate atom pairs within the van der Waals distance (sum of atomic radii plus a 0.4 Å tolerance) in line 635.

l. 615 italicize p

It is corrected accordingly.

Fig 1D This presentation is less informative. Perhaps we could get scores in the table? I assume these are the only ones on the list because of a cutoff used: was it a Z-score, a certain log2FC threshold, etc?

We appreciate the suggestion and have updated the figure accordingly. The hits in figure 1D represent those peptides that showed detectable and reliable modification of tyrosine residues with our labelling reagent (biotin-tyramide). We have included a table to display the values.

Fig 2 Preferred to use colorblind-safe colors

We thank the reviewer for this suggestion. We have revised the figures to use colorblind-safe color schemes in all cases where red and green channels were previously combined, specifically in Fig. 1, Fig. 2, and Fig. 4.

Fig 6 B-C It would be useful to have a transparent isosurface overlay so we know the extent to which these side-chain-level interactions are supported by density vs. inferred by overall chain placement

Figure 6 has been modified accordingly.

Extended Fig 7 C-D At the reported resolution, it would be more scientifically meaningful to use an atom depiction instead of a ribbon depiction.
7F Why are both transparent densities the same base color?

We thank the reviewer for the suggestions. The figures, which is now in Extended Fig 8, are updated accordingly.

I would recommend adding the 2.23Å NU-refinement map as an "Additional map" to the EMDB deposition because of the ongoing uncertainties around how to interpret 3DFlex densities.

We have added the 2.23 Å NU-refinement maps (sharpened and unsharpened) to the EMDB deposition.

Take a look at the Asn/Gln/His flips on the deposited models.

We thank the reviewer for this suggestion. We have modified the models accordingly, resulting in a significant reduction in the number of listed residues. The updated models have been deposited in the PDB entries.

Nature Communications manuscript NCOMMS-26-021762A

REVIEWER COMMENTS (black font)

Point by point response (blue)

Reviewer #1 (Remarks to the Author):

The authors sufficiently addressed all my concerns and comments. The new edits have significantly improved the manuscript by making the findings easier to interpret and by providing additional context when discussing the results. The additional supplementary figures have significantly improved the presentation of the results. This will be of great interest for the structural-, clinical- and parvovirus fields. I have no further comments.

We thank the Reviewer for the positive evaluation of our work and for the constructive feedback throughout the review process. We are pleased that the revised manuscript and additional supplementary data improved the clarity and presentation of the study.

Reviewer #2 (Remarks to the Author):

In the discussion of the revised manuscript, there is only text and citations. Please discuss in depth the "TfR1 entry factor " shared by those viruses (non-erythroid cells) and B19V (erythroid cells).

We thank the Reviewer for this comment. We believe this point is now addressed in the revised Discussion, where we discuss TfR1 usage by multiple viruses and emphasize that, unlike other TfR1-dependent viruses infecting non-erythroid cells, B19V requires an erythroid-specific cellular context for productive VP1u engagement despite broad TfR1 expression.
